# Establishing the Role of Iridoids as Potential Kirsten Rat Sarcoma Viral Oncogene Homolog G12C Inhibitors Using Molecular Docking; Molecular Docking Simulation; Molecular Mechanics Poisson–Boltzmann Surface Area; Frontier Molecular Orbital Theory; Molecular Electrostatic Potential; and Absorption, Distribution, Metabolism, Excretion, and Toxicity Analysis

**DOI:** 10.3390/molecules28135050

**Published:** 2023-06-28

**Authors:** Mubarak A. Alamri, Abdullah S. Alawam, Mohammed Merae Alshahrani, Sarkar M. A. Kawsar, Prinsa, Supriyo Saha

**Affiliations:** 1 Department of Pharmaceutical Chemistry, College of Pharmacy, Prince Sattam Bin Abdulaziz University, Al-Kharj 11942, Saudi Arabia; m.alamri@psau.edu.sa; 2 Department of Biology, College of Science, Imam Mohammad Ibn Saud Islamic University, Riyadh 11623, Saudi Arabia; asalawam@imamu.edu.sa; 3 Department of Clinical Laboratory Sciences, Faculty of Applied Medical Sciences, Najran University, 1988, Najran 61441, Saudi Arabia; mmalshahrani@nu.edu.sa; 4 Laboratory of Carbohydrate and Nucleoside Chemistry, Department of Chemistry, Faculty of Science, University of Chittagong, Chittagong 4331, Bangladesh; akawsarabe@yahoo.com; 5 Siddhartha Institute of Pharmacy, Near IT-Park, Sahastradhara Road, Dehradun 248001, Uttarakhand, India; 6 Uttaranchal Institute of Pharmaceutical Sciences, Uttaranchal University, Premnagar, Dehradun 248007, Uttarakhand, India

**Keywords:** KRAS G12C, Iridoids, Sotorasib, molecular docking, MD simulation, MM/PBSA, FMO, MEP, ADMET

## Abstract

The RAS gene family is one of the most frequently mutated oncogenes in human cancers. In KRAS, mutations of G12D and G12C are common. Here, 52 iridoids were selected and docked against 8AFB (KRAS G12C receptor) using Sotorasib as the standard. As per the docking interaction data, 6-*O*-*trans*-*p*-coumaroyl-8-*O*-acetylshanzhiside methyl ester (dock score: −9.9 kcal/mol), 6′-*O*-*trans*-*para*-coumaroyl geniposidic acid (dock score: −9.6 kcal/mol), 6-*O*-*trans*-cinnamoyl-secologanoside (dock score: −9.5 kcal/mol), Loganic acid 6′-*O*-beta-d-glucoside (dock score: −9.5 kcal/mol), 10-*O*-succinoylgeniposide (dock score: −9.4), Loganic acid (dock score: −9.4 kcal/mol), and Amphicoside (dock score: −9.2 kcal/mol) showed higher dock scores than standard Sotorasib (dock score: −9.1 kcal/mol). These common amino acid residues between iridoids and complexed ligands confirmed that all the iridoids perfectly docked within the receptor’s active site. The 100 ns MD simulation data showed that RMSD, RMSF, radius of gyration, and SASA values were within range, with greater numbers of hydrogen bond donors and acceptors. MM/PBSA analysis showed maximum binding energy values of −7309 kJ/mol for 6-*O*-*trans*-*p*-coumaroyl-8-*O*-acetylshanzhiside methyl ester. FMO analysis showed that 6-*O*-*trans*-*p*-coumaroyl-8-*O*-acetylshanzhiside methyl ester was the most likely chemically reactive molecule. MEP analysis data highlighted the possible electrophilic and nucleophilic attack regions of the best-docked iridoids. Of all the best-docked iridoids, Loganic acid passed Lipinski, Pfizer, and GSK filters with a similar toxicity profile to Sotorasib. Thus, if we consider these iridoids to be KRAS G12C inhibitors, they will be a boon to mankind.

## 1. Introduction

The Kirsten rat sarcoma viral oncogene homolog (KRAS) is an oncogene with a high mutation rate and is associated with several aggressive and often fatal cancers, including pancreatic ductal adenocarcinoma, non-small-cell lung cancer, and colorectal cancer. Among KRAS mutations, G12 mutations are the most common, accounting for around 89% of cases. Within G12 mutations, G12D is the most prevalent (36%), followed by G12V (23%) and G12C (14%). G13 mutations make up about 9% of cases, and Q61 mutations are the least common at around 1%. Different cancer types exhibit varying frequencies of specific KRAS mutation subtypes. For example, G12D plays a major role in pancreatic ductal adenocarcinoma (PDAC), while G12C is the most common subtype in non-small-cell lung cancer (NSCLC), accounting for about 13% of cases [1]. Targeted therapies have significantly improved survival outcomes for patients with certain genetic alterations, such as epidermal growth factor receptor (EGFR)-sensitive mutations or anaplastic lymphoma kinase (ALK) gene fusions [2]. However, targeting KRAS mutations has been challenging, and until recently, there were no effective strategies specifically targeting KRAS mutations [3]. Sotorasib (AMG510) is a KRAS (G12C) inhibitor that was recently approved as the first targeted therapy for patients with KRAS (G12C) mutations. However, it is important to note that this drug is specific to the G12C subtype. Given the inherent characteristics of KRAS proteins, structures, and binding properties, directly targeting KRAS has been difficult [4]. As a result, many efforts have focused on indirect strategies to target KRAS, including inhibiting downstream signaling effectors, utilizing epigenetic approaches like telomerase inhibitors and RNA interference, and exploring synthetic lethality approaches such as cyclin-dependent kinase inhibitors. Other investigational KRAS (G12C) inhibitors, such as MRTX849 (Adagrasib), have shown promise in clinical trials but are not yet approved for use in the market [5]. The development of effective therapies targeting KRAS mutations remains an active area of research, and advancements in this field hold great potential for improving treatment options for patients with KRAS-mutated cancers [6]. Inhibiting farnesyl transferase enzyme, the enzyme responsible for the post-translational modification of KRAS, and targeting downstream effectors of KRAS have been strategies explored in the past [7]. However, these approaches have not been successful in clinical trials over the past three decades. More recently, a breakthrough in targeting the KRAS-G12C mutation was achieved through the development of covalent inhibitors [8]. These inhibitors selectively form a covalent bond with cysteine 12 within the switch-II pocket of the KRAS-G12C protein, locking it in an inactive state and halting cell proliferation [9]. Sotorasib (AMG510) and Adagrasib (MRTX849) are examples of covalent inhibitors that have shown promise in clinical trials specifically targeting the KRAS-G12C mutation. Interestingly, it has been observed that these inhibitors do not directly affect the PI3K signaling pathway. This suggests that upstream pathways independent of KRAS-G12C play a role in facilitating the activation of PI3K, providing a possible explanation for the development of resistance to KRAS-G12C inhibitors. Additionally, studies have shown that Sotorasib exhibits enhanced anticancer activity in immunocompetent mice compared with immunodeficient mice [10]. This highlights the potential role of KRAS-G12C inhibitors in triggering a pro-inflammatory tumor microenvironment, leading to increased T-cell infiltration and activation in response to immune checkpoint inhibitors like anti-PDCD1/PD-1 antibodies. Clinical trials (NCT04185883 and NCT03785249) are currently underway to evaluate the combination of KRAS-G12C inhibitors (Adagrasib or Sotorasib) with pembrolizumab (an anti-PDCD1/PD-1 antibody) in patients with advanced/metastatic solid tumors harboring the KRAS-G12C mutation [11]. Iridoids are a class of natural cyclopentanepyran monoterpenes that are found abundantly in dicotyledonous plants from various families such as *Scrophulariaceae, Pyrolaceae, Oleaceae, Labiatae, Rubiaceae*, and *Gentianaceae*. They can be classified into different subtypes based on their chemical structure: iridoid glycosides, secoiridoid glycosides, non-glycosidic iridoids, and bis-iridoids. Iridoid glycosides, such as geniposide, loganin, acetylbarlerin, deacetylasperulosidic acid, and brasoside, have been studied for their potential anticancer effects [12,13]. Secoiridoid glycosides, including swertiamarine, gentiopicroside, qinjiaoside A, qinjiaoside B, sweroside, and oleuropein, are another subtype of iridoids [14]. Although their specific effects on cancer growth have not been extensively studied, these compounds have demonstrated various biological activities, including anti-inflammatory and antioxidant properties. Non-glycosidic iridoids, such as acevaltrate and valtrate, have been investigated for their potential anticancer effects [15]. Their specific mechanisms of action and effects on cancer growth are still being explored. Bis-iridoids, including cantleyoside, laciniatoside I–II, and sylvestroside I, are a less well-studied subtype of iridoids in the context of cancer research [16]. Overall, iridoids, particularly iridoid glycosides, have shown promise in inhibiting cancer growth by inducing cell cycle arrest, regulating apoptosis-related signaling pathways, and suppressing MMP expression and activity. Further research is needed to fully understand their mechanisms of action and their potential as therapeutic agents against cancer. In this manuscript, we investigated the role of iridoids as KRAS G12C inhibitors using various computational techniques such as molecular docking, MD simulation, MM/PBSA, FMO theory, and MEP analysis.

## 2. Results

### 2.1. Molecular Docking Interaction Data

**Figure 1:**
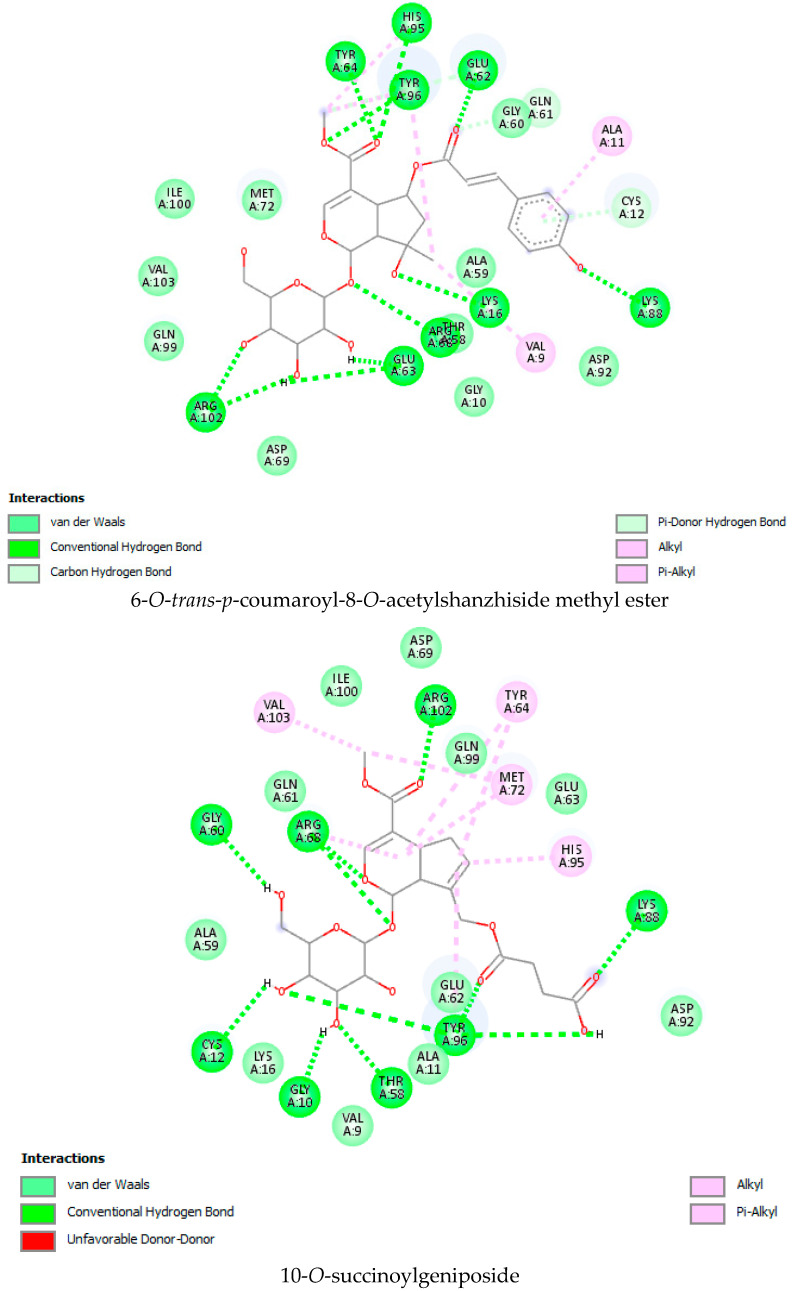

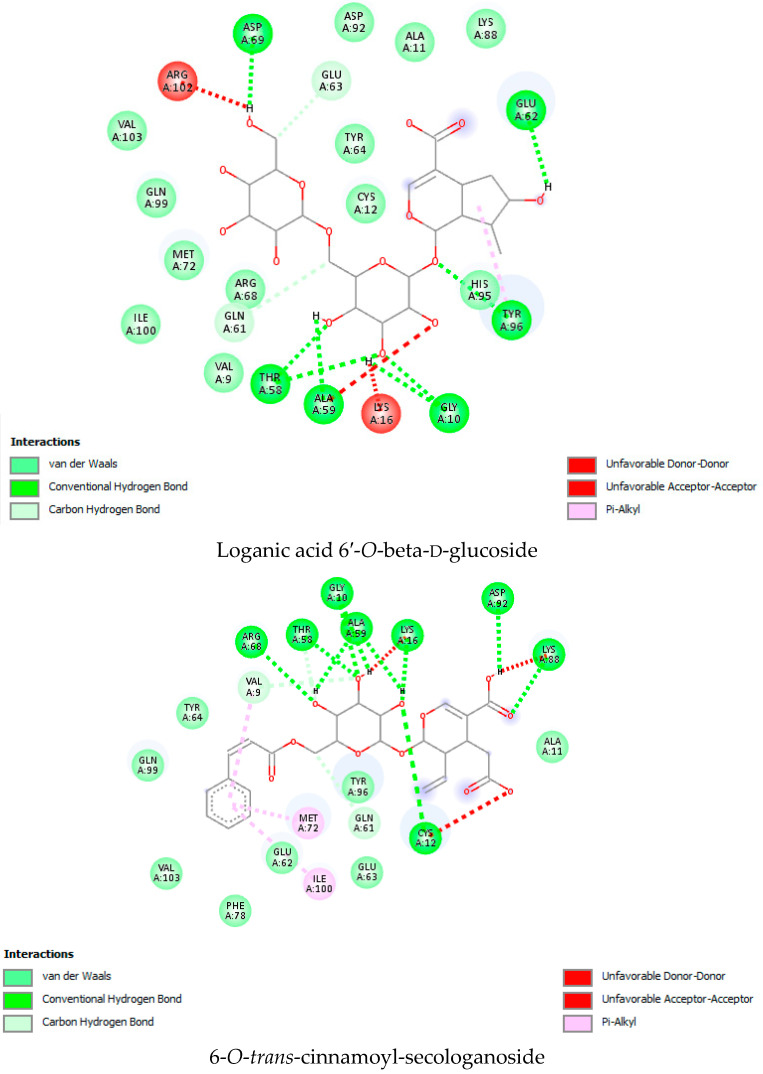

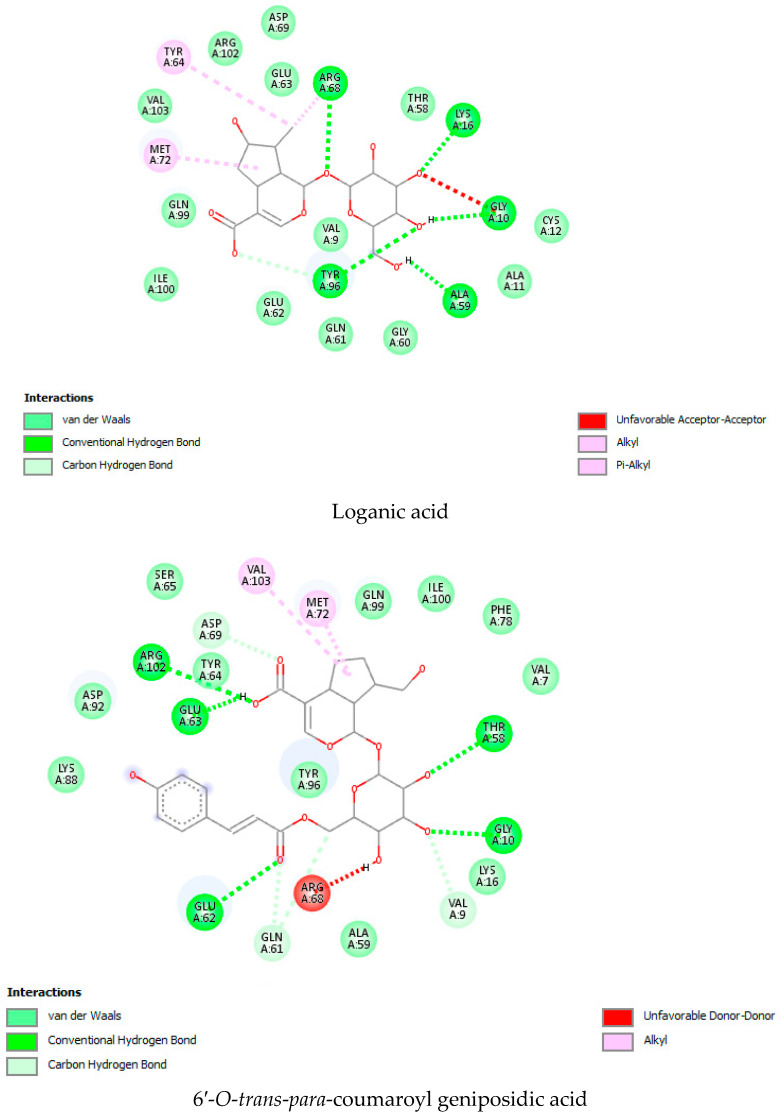

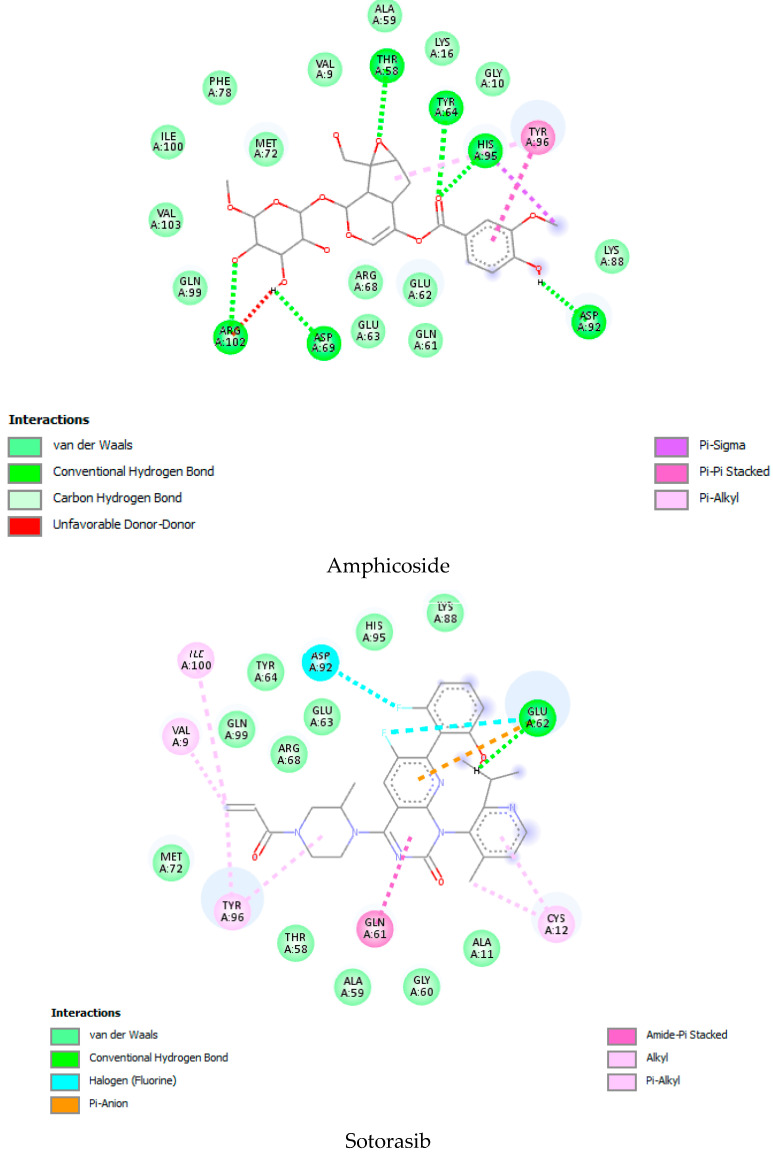
Molecular docking interaction data for 6-*O*-*trans*-*p*-coumaroyl-8-*O*-acetylshanzhiside methyl ester, 10-*O*-succinoylgeniposide, Loganic acid 6′-*O*-beta-d-glucoside, 6-*O*-*trans*-cinnamoyl-secologanoside, Loganic acid, 6′-*O*-*trans*-*para*-coumaroyl geniposidic acid, Amphicoside, and Sotorasib.

As per the drug discovery studio data, the co-crystallized ligand (LXD) of the 8AFB receptor connected with CYS 12, GLU 63, ASP 69, ASP 92, and HIS 95 via hydrogen bond interactions. Also, PRO 34, GLY 60, GLN 61, ALA 59, GLU 62, ARG 68, GLN 99, TYR 96, ARG 102, ILE 100, and VAL 103 amino acids interacted with LXD via hydrophobic interactions (Figure S1). In this study, 52 iridoids (Table S1) from different categorieswere selected and docked with the 8AFB receptor. As per the molecular docking interaction study data, reference-standard Sotorasib showed a docking score of −9.1 kcal/mol, and only seven molecules showed higher docking energy scores than Sotorasib. Among them, 6-*O*-*trans*-*p*-coumaroyl-8-*O*-acetylshanzhiside methyl ester showed a maximum dock score of −9.9 kcal/mol, followed by 6′-*O*-*trans*-*para*-coumaroylgeniposidic acid (dock score: −9.6 kcal/mol), 6-*O*-*trans*-cinnamoyl secologanoside (dock score: −9.5 kcal/mol), Loganic acid 6′-*O*-beta-d-glucoside (dock score: −9.5 kcal/mol), 10-*O*-succinoylgeniposide (dock score: −9.4 kcal/mol),Loganic acid (dock score: −9.4 kcal/mol),and Amphicoside(dock score: −9.2 kcal/mol).Also, after redocking LXD, a −11.7 kcal/mol dock score was observed (Figure 1). All the molecular docking interaction data are in Table 1 [17].

### 2.2. MD Simulation Data

**Figure 2:**
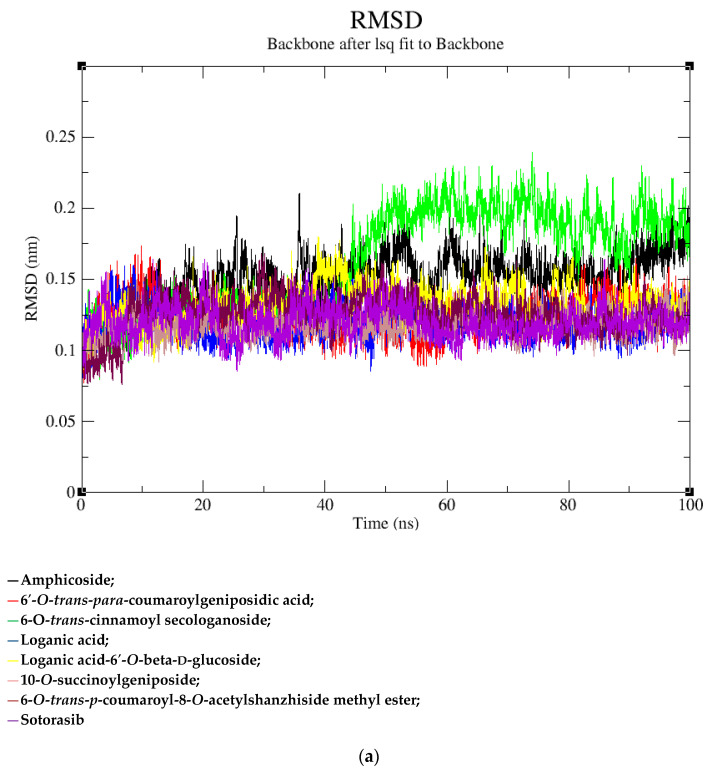

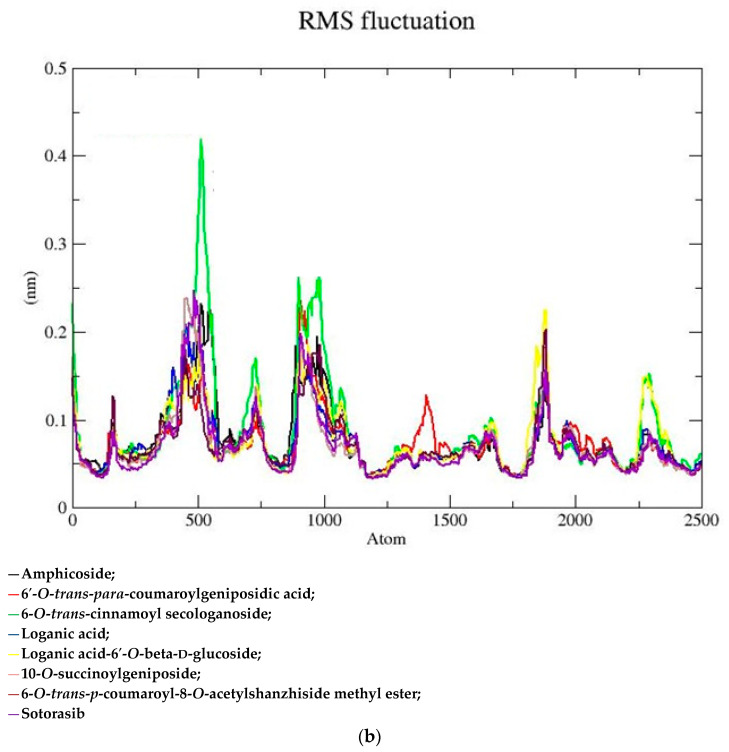

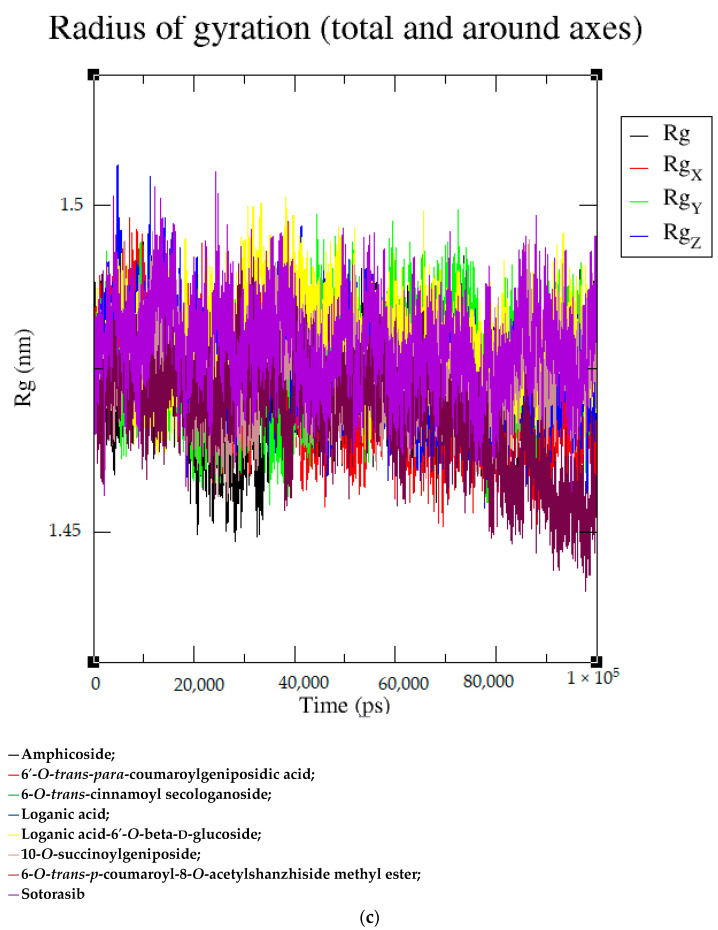

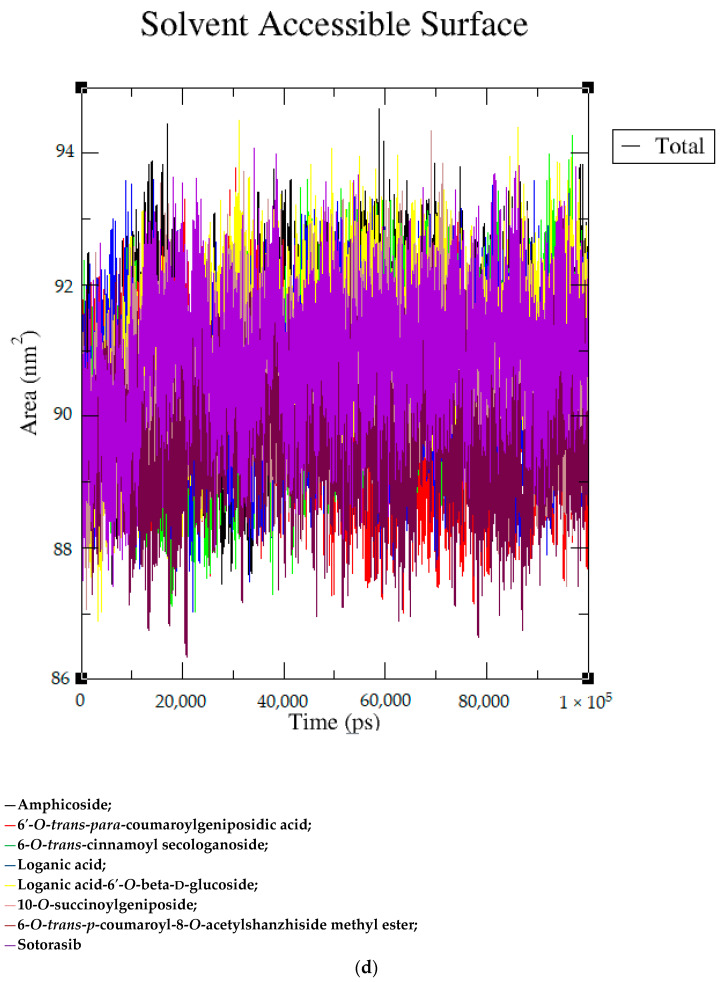

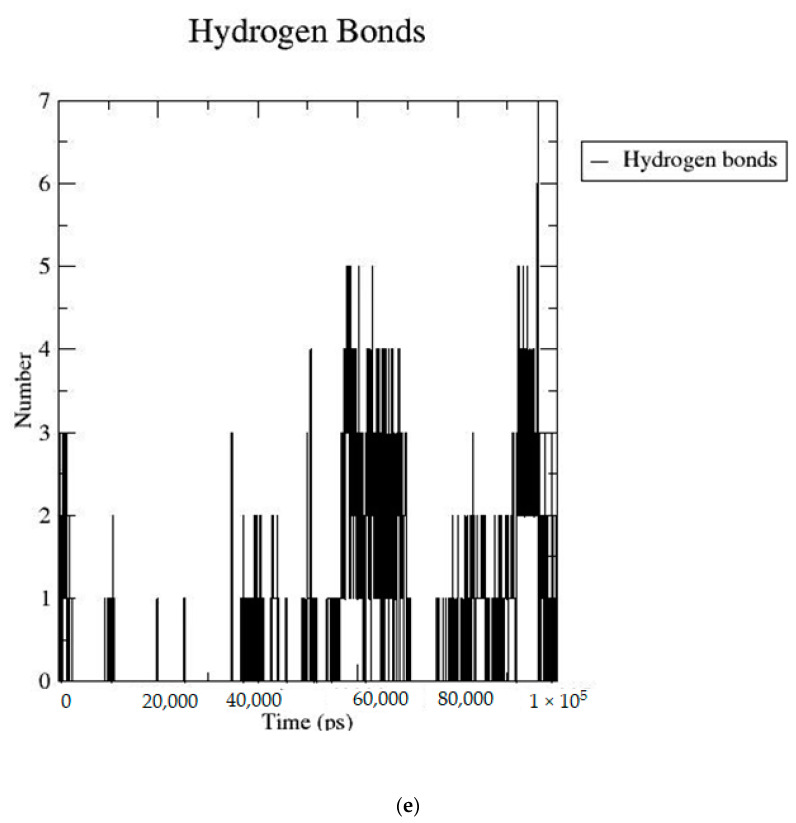

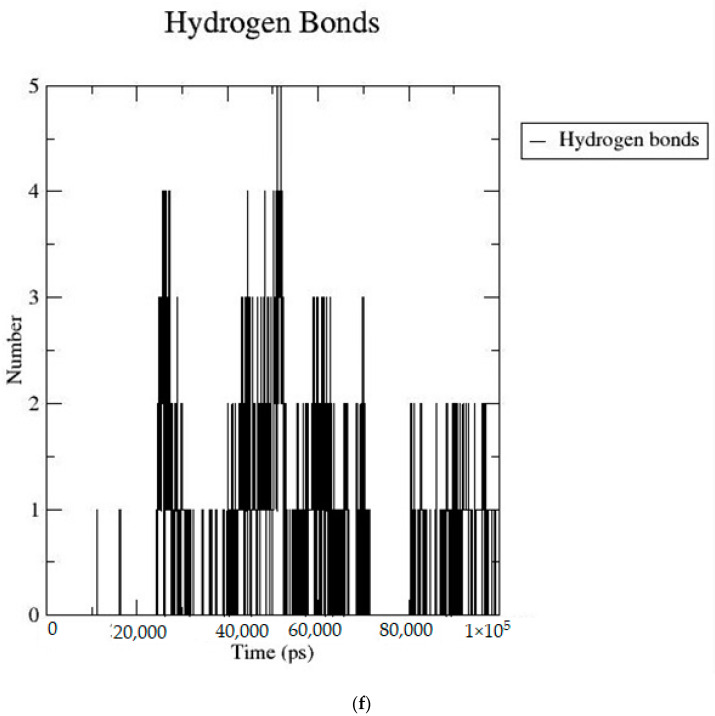

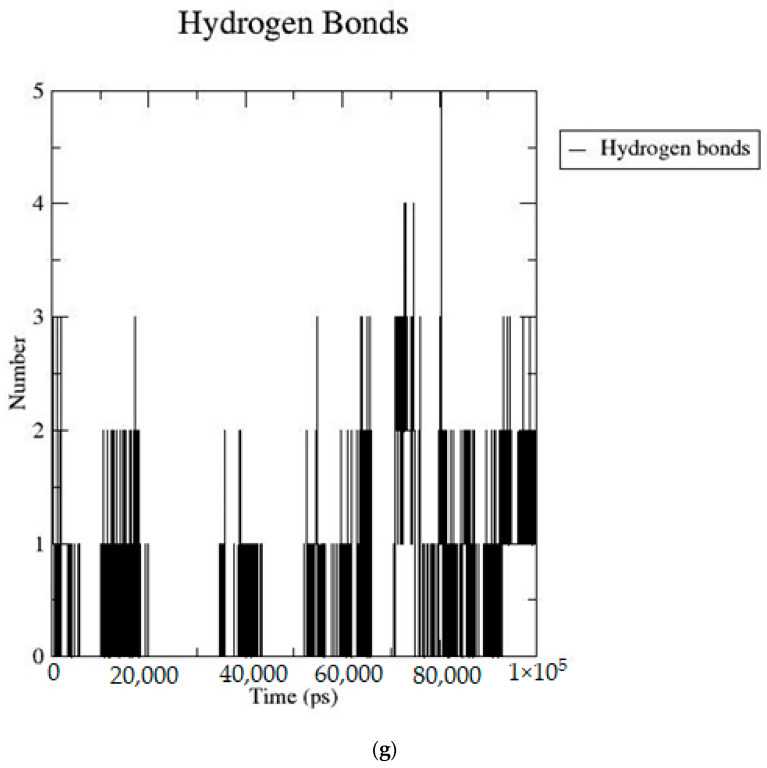

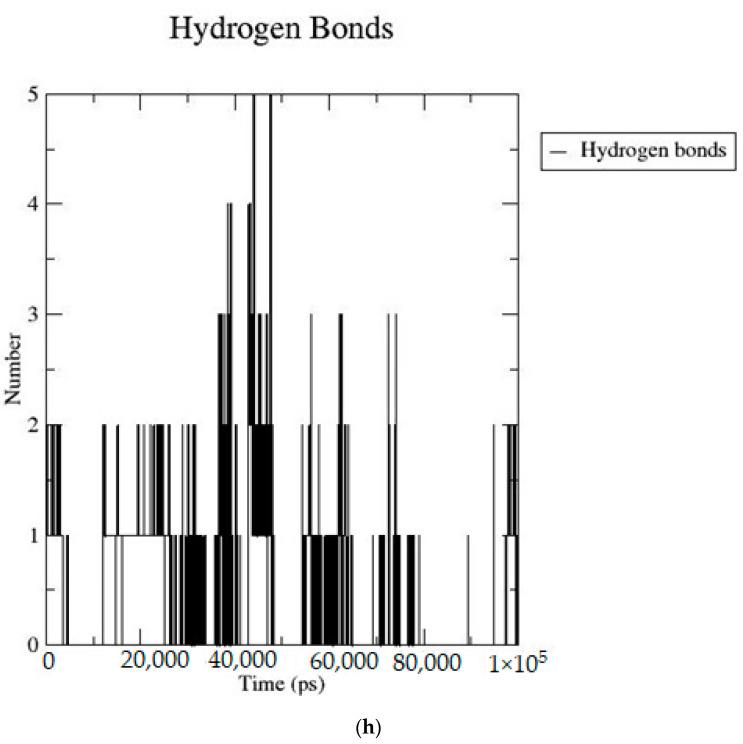

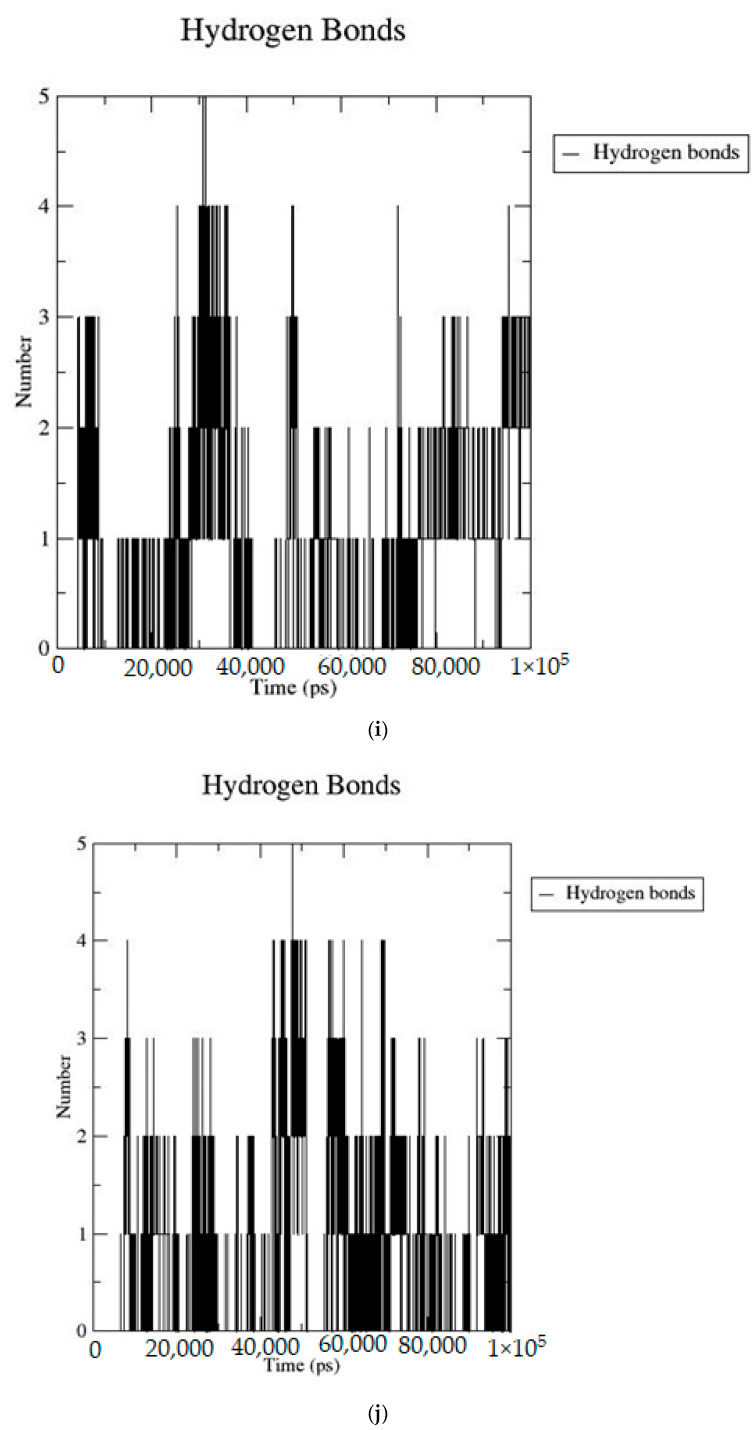

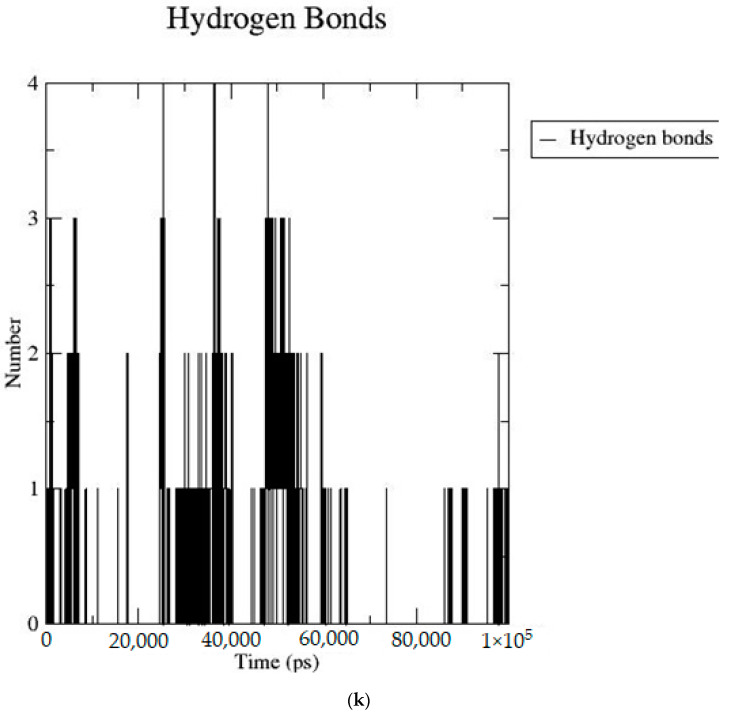

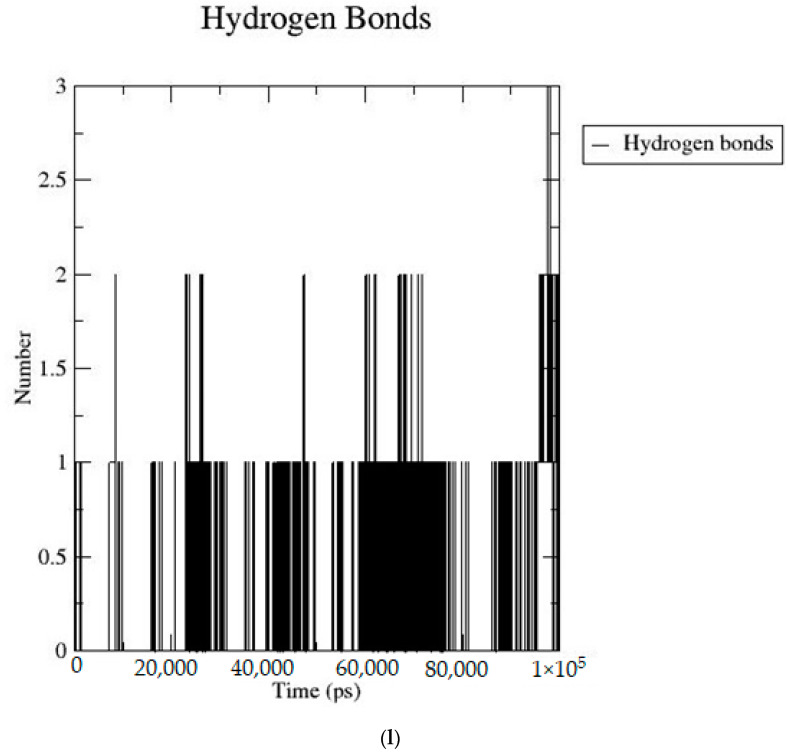
(**a**–**l**) MD simulation data for Iridoids and Sotorasib with 8AFB.

Based on the docking scores, 6-*O*-*trans*-*p*-coumaroyl-8-*O*-acetylshanzhiside methyl ester, 10-*O*-succinoylgeniposide, Loganic acid 6′-*O*-beta-d-glucoside, 6-*O*-*trans*-cinnamoyl-secologanoside, Loganic acid, 6′-*O*-*trans*-*para*-coumaroylgeniposidic Acid, Amphicoside, and Sotorasib were selected for the 100 ns MD simulation study. As per the molecular dynamic simulation data from the iridoids and 8AFB receptor, the average RMSD values for 6-*O*-*trans*-*p*-coumaroyl-8-*O*-acetylshanzhiside methyl ester, 10-*O*-succinoyl geniposide, Loganic acid 6′-*O*-beta-d-glucoside, 6-*O*-*trans*-cinnamoyl secologanoside, Loganic acid, 6-*O*-*trans*-coumaroylgeniposidic acid, Amphicoside, and Sotorasib were 0.12, 0.11,0.13,0.16,0.11, 0.12, 0.15, and 0.11, respectively (Figure 2a). In the RMSF data, some observable fluctuations were identified near 500, 1000, and 1800 atoms, with maximum fluctuations within 0.25 nm, except 6-*O*-*trans*-cinnamoyl secologanoside, which showed a sudden surge in values up to 0.42 nm (Figure 2b). The radius of gyration values for 6-*O*-*trans*-*p*-coumaroyl-8-*O*-acetylshanzhiside methyl ester, 10-*O*-succinoyl geniposide, Loganic acid 6′-*O*-beta-d-glucoside, 6-*O*-*trans*-cinnamoyl secologanoside, Loganic acid, 6-*O*-*trans*-coumaroyl geniposidic acid, Amphicoside, and Sotorasib were within ranges of (1.44–1.49), (1.45–1.49), (1.45–1.50), (1.45–1.49), (1.45–1.50), (1.45–1.50), (1.44–1.49), and (1.14–1.27), respectively (Figure 2c). The average SASA values of 6-*O*-*trans*-*p*-coumaroyl-8-*O*-acetylshanzhiside methyl ester, 10-*O*-succinoyl geniposide, Loganic acid 6′-*O*-beta-d-glucoside, 6-*O*-*trans*-cinnamoyl secologanoside, Loganic acid, 6-*O*-*trans* coumaroyl geniposidic acid, Amphicoside, and Sotorasib were 89.59 nm^2^, 90.60 nm^2^, 91.03 nm^2^, 90.55 nm^2^, 90.48 nm^2^, 89.97 nm^2^, 90.91, and 90.90 nm^2^, respectively (Figure 2d). The hydrogen bond analysis of 6-*O*-*trans*-*p*-coumaroyl-8-*O*-acetylshanzhiside methyl ester (Figure 2e), 10-*O*-succinoyl geniposide (Figure 2f), Loganic acid 6′-*O*-beta-d-glucoside (Figure 2g), 6-*O*-*trans*-cinnamoyl secologanoside (Figure 2h), Loganic acid (Figure 2i), 6-*O*-*trans* coumaroyl geniposidic acid (Figure 2j), Amphicoside (Figure 2k), and Sotorasib (Figure 2l) showed that 251, 250, 254, 250, 251, 251, 250, and 246 hydrogen bond donors and 497, 497, 499, 496, 494, 496, 497, and 493 hydrogen bond acceptors were present in the interactions. The coulombic short-range:protein–ligand energies (kJ/mol) for 6-*O*-*trans*-*p*-coumaroyl-8-*O*-acetylshanzhiside methyl ester, 10-*O*-succinoyl geniposide, Loganic acid 6′-*O*-beta-d-glucoside, 6-*O*-*trans*-cinnamoyl secologanoside, Loganic acid, 6-*O*-*trans* coumaroyl geniposidic acid, Amphicoside, and Sotorasib were −30.52, −22.47, −21.20, −35.74, −41.27, −35.387, −13.98, and −21.32 respectively. The Lennard–Jonesshort-range:protein–ligand energies of the molecules were −24.00 kJ/mol, −47.71 kJ/mol, −30.30 kJ/mol, −27.82 kJ/mol, −45.18, −36.75, −25.85, and −106.351, respectively [18].

### 2.3. MM/PBSA Analysis Data

6-*O*-*trans*-*p*-coumaroyl-8-*O*-acetylshanzhiside methyl ester, 10-*O*-succinoylgeniposide, Loganic acid 6′-*O*-beta-d-glucoside, 6-*O*-*trans*-cinnamoyl-secologanoside, Loganic acid, 6′-*O*-*trans*-*para*-coumaroyl geniposidic acid, Amphicoside, and Sotorasib were selected for MM/PBSA analysis. The MM/PBSA analysis data showed maximum binding energy by 6-*O*-*trans*-*p*-coumaroyl-8-*O*-acetylshanzhiside methyl ester (−7.309 kJ/mol), followed by 6′-*O*-*trans*-*para*-coumaroyl geniposidic acid (−6.153 kJ/mol) and 6-*O*-*trans*-cinnamoyl-secologanoside (−4.971 kJ/mol) (Table 2) [19].

### 2.4. Frontier Molecular Orbital (FMO) Analysis Data

**Figure 3:**
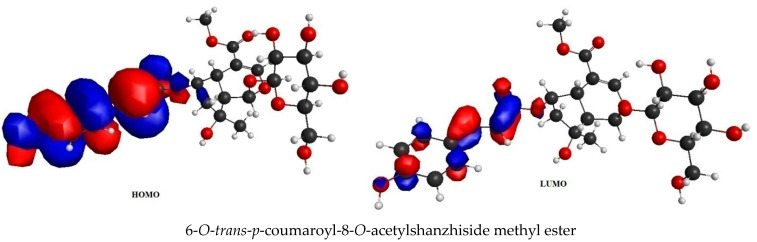

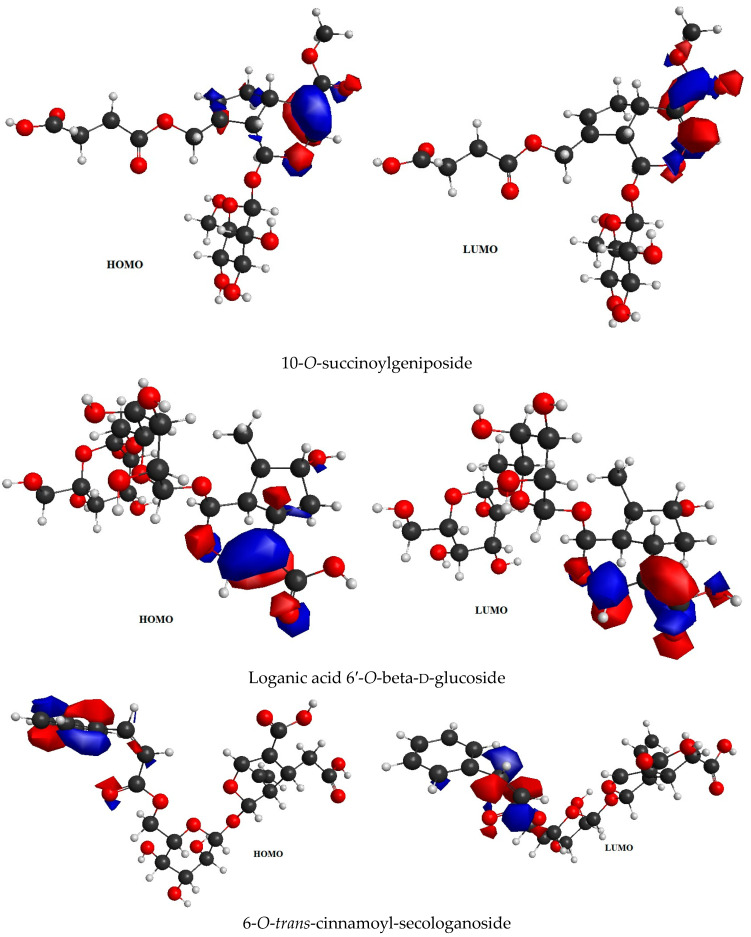

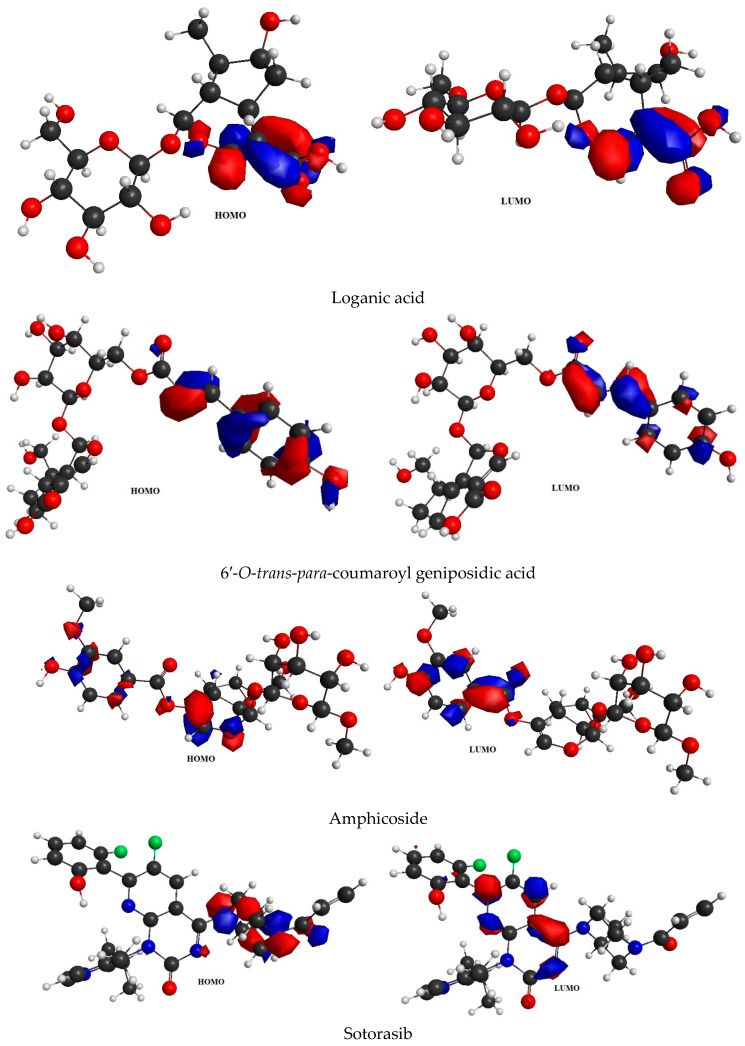
FMO analysis data for 6-*O*-*trans*-*p*-coumaroyl-8-*O*-acetylshanzhiside methyl ester, 10-*O*-succinoylgeniposide, Loganic acid 6′-*O*-beta-d-glucoside, 6-*O*-*trans* cinnamoyl-secologanoside, Loganic acid, 6′-*O*-*trans*-*para*-coumaroyl geniposidic acid, Amphicoside, and Sotorasib.

6-*O*-*trans*-P-coumaroyl-8-*O*-acetylshanzhiside methyl ester, 10-*O*-succinoylgeniposide, Loganic acid 6′-*O*-beta-d-glucoside, 6-*O*-*trans*-cinnamoyl-secologanoside, Loganic acid, 6′-*O*-*trans*-*para*-coumaroyl geniposidic acid, Amphicoside, and Sotorasib were selected for frontier molecular orbital analysis. The HOMO orbital energy (eV) values of 6-*O*-*trans*-*p*-coumaroyl-8-*O*-acetylshanzhiside methyl ester, 10-*O*-succinoyl geniposide, Loganic acid 6′-*O*-beta-d-glucoside, 6-*O*-*trans*-cinnamoyl secologanoside, Loganic acid, 6-*O*-*trans*coumaroyl geniposidic acid, Amphicoside, and Sotorasib were −6.44, −6.63, −6.83, −6.55, −6.80, −6.34, −6.28, and −6.34, respectively. The LUMO orbital energy (eV) values of 6-*O*-*trans*-*p*-coumaroyl-8-*O*-acetylshanzhiside methyl ester, 10-*O*-succinoyl geniposide, Loganic acid 6′-*O*-beta-d-glucoside, 6-*O*-*trans*-cinnamoyl secologanoside, Loganic acid, 6-*O*-*trans* coumaroyl geniposidic acid, Amphicoside, and Sotorasib were −2.14, −1.06, −1.41, −1.57, −1.25, −1.95, −1.49, and −2.74, respectively. The energy gap between the HOMO and LUMO orbitals indicates the chemical strength and reactivity of the molecules [20]. The minimum energy gap between the HOMO and LUMO orbitals was observed with Sotorasib (standard), 6-*O*-*trans*-*p*-coumaroyl-8-*O*-acetylshanzhiside methyl ester, and 6′-*O*-*trans*-*para*-coumaroyl geniposidic acid at 3.6 eV, 4.3 eV, and 4.39 eV, respectively by considering B3LYP/6-31+ G(d,p) basis sets (Table 3 and Figure 3).

### 2.5. Molecular Electrostatic Potential (MEP) Data

**Figure 4:**
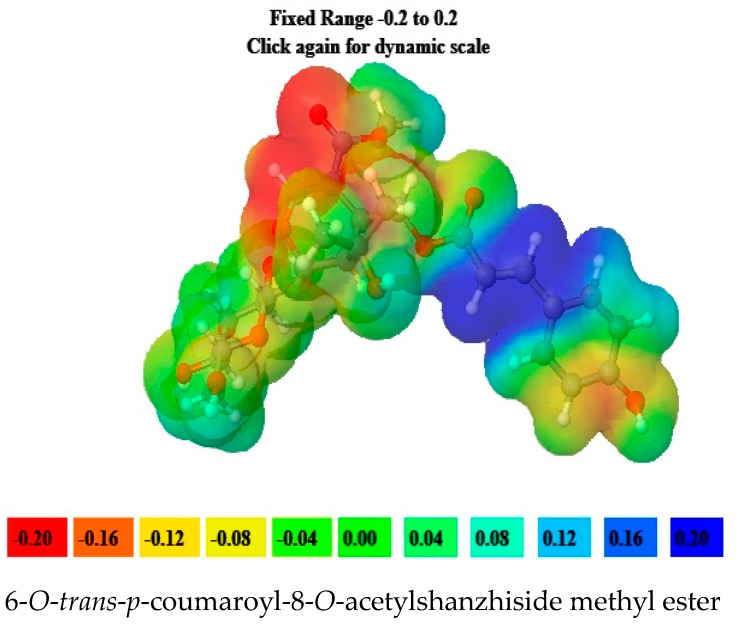

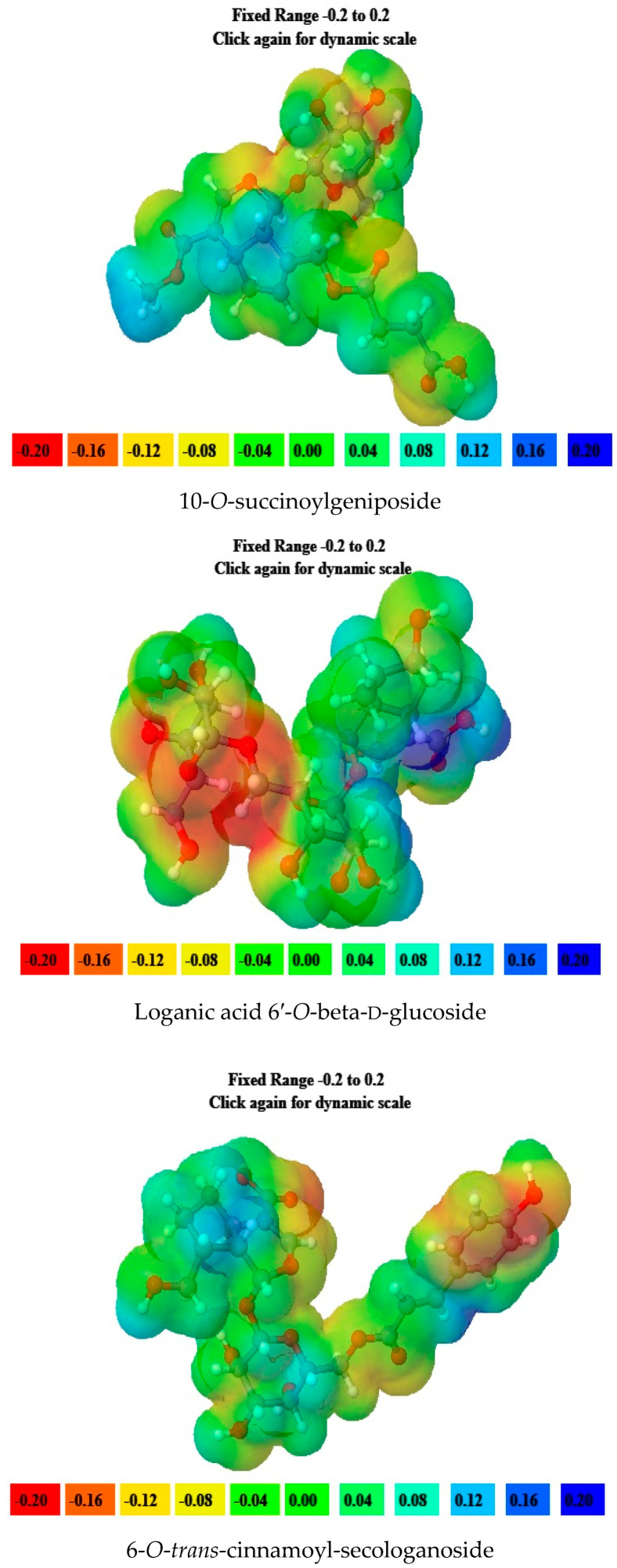

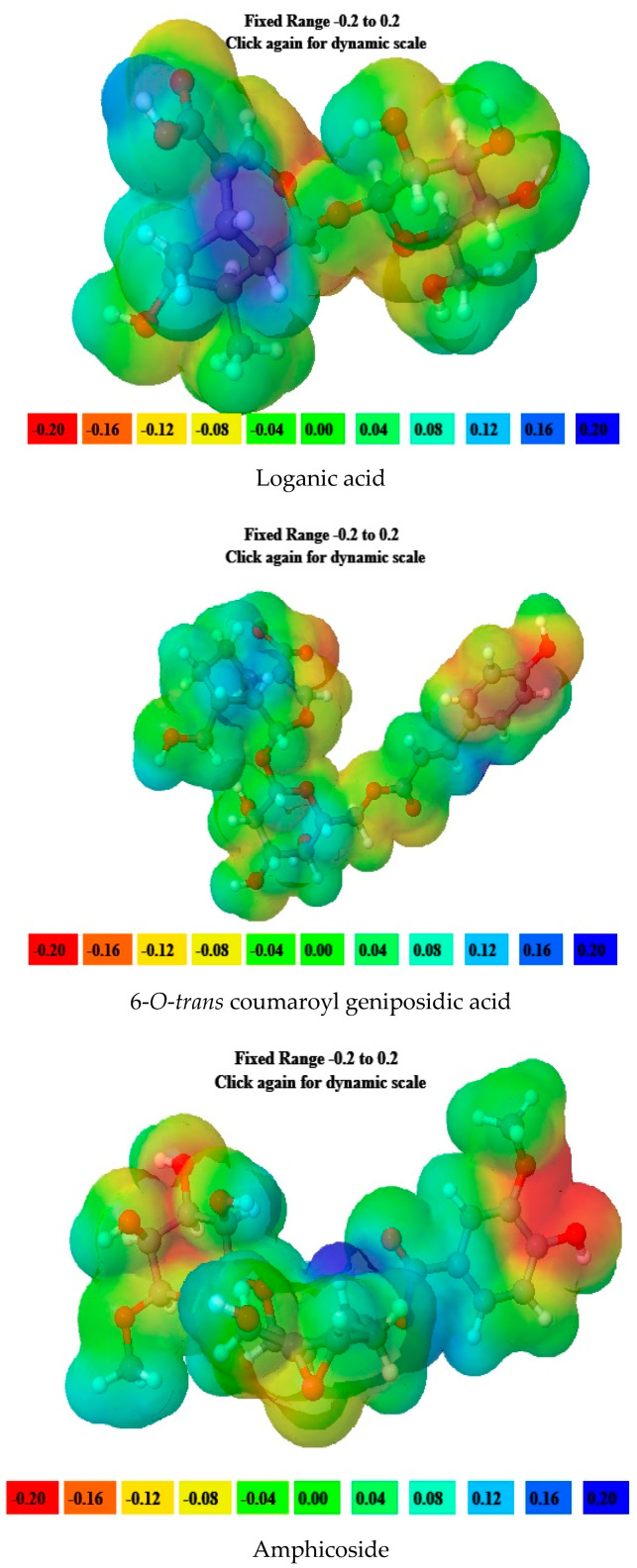

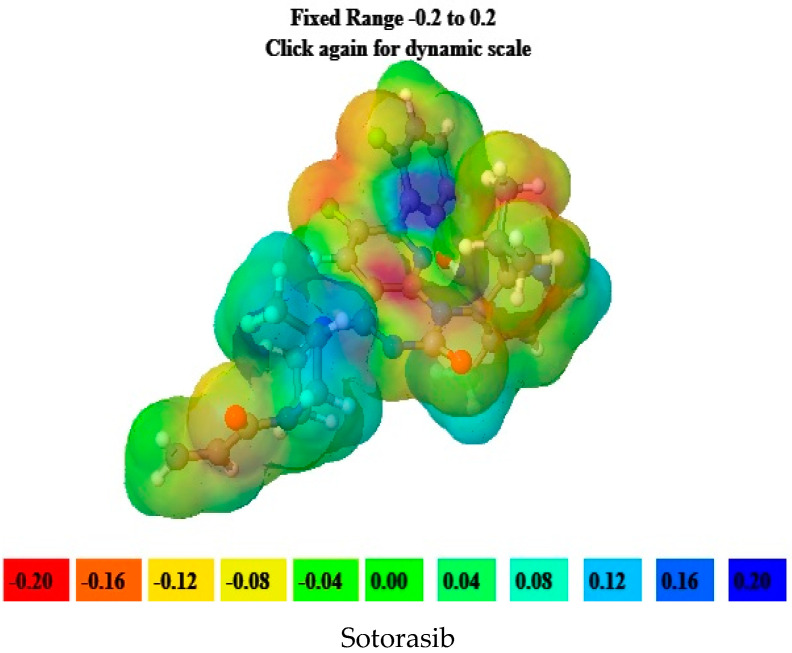
MEP analysis data for 6-*O*-*trans*-*p*-coumaroyl-8-*O*-acetylshanzhiside methyl ester, 10-*O*-succinoylgeniposide, Loganic acid 6′-*O*-beta-d-glucoside, 6-*O*-*trans* cinnamoyl-secologanoside, Loganic acid, 6′-*O*-*trans*-*para*-coumaroyl geniposidic acid, Amphicoside, and Sotorasib.

6-*O*-*trans*-*p*-coumaroyl-8-*O*-acetylshanzhiside methyl ester, 10-*O*-succinoylgeniposide, Loganic acid 6′-*O*-beta-d-glucoside, 6-*O*-*trans*-cinnamoyl-secologanoside, Loganic acid, 6′-*O*-*trans*-*para*-coumaroyl geniposidic acid, Amphicoside, and Sotorasib were selected for molecular electrostatic potential analysis. As per MEP, the blue region corresponds with probable nucleophilic attack regions and the red region corresponds with probable electrophilic attack regions. In 6-*O*-*trans*-*p*-coumaroyl-8-*O*-acetylshanzhiside methyl ester, the most negative red-colored portion was placed in the (2E)-3-hydroxyprop-2-enoate group, and the most positive blue-colored zone was focused on the C=C-C=C group of the 4-hydroxy cinnamoyl group. In 10-*O*-succinoyl geniposide, a slightly negative red-colored portion was placed in the hydroxyl groups ofglucose moiety, and a slightly positive blue-colored zone was focused on cyclopentane in the methyl 3a,4,5,6,7,7a-hexahydro-1H-indene-7-carboxylate group. In the case of Loganic acid 6′-*O*-beta-d-glucoside, the most negative red-colored portion was placed in the glucose moiety, and the positive blue-colored region was focused on the junction of the 6-hydroxy-7-methyl-1,4a,5,6,7,7a-hexahydrocyclopenta[c]pyran-4-carboxylic acid system. The red and blue colors of 6-*O*-*trans*-cinnamoyl secologanoside focused on oxygen atoms in the O-C=O and-COOH groups and the glucopyranose moiety, respectively. In the case of Loganic acid, a red-colored zone was focused on the hydroxyl group andthe carboxylic acid group or ether oxygen, and the blue-colored zone was focused on the carbon reach group of 6-hydroxy-7-methyl-1,4a,5,6,7,7a-hexahydrocyclopenta[c]pyran-4-carboxylic acid. The negative red-colored zone of 6-*O*-*trans* coumaroyl geniposidic acid focused on the 4-hydroxy group of the cinnamoyl ring, and the blue-colored region was focused on the HC=CH group. In the case of Amphicoside, the negative red-colored region was observed in the hydroxyl and methoxy group, and the positive blue-colored region was focused on the junction of the 1a,1b,2,5a,6,6a-hexahydrooxireno[4,5]cyclopenta[1,2-c]pyran group. In the case of the reference standard, Sotorasib, the maximum molecule was covered with blue-colored positive regions (the nitrogen part of 6-fluoropyrido[2,3-d]pyrimidin-2(1H)-one and piperazine) (Figure 4) [21].

### 2.6. ADMET (Absorption, Distribution, Metabolism, Excretion, Toxicity) Data

The ADMET analysis data of all the selected iridoids showed that, except for Sotorasib, all the iridoids were P glycoprotein non inhibitors. Molecules with a Log S value between −4 and 0.5 Log mol/L were considered to have good aqueous solubility. Except for 10-Isovaleroyl-dihydropenstemide and Sotorasib, the rest of the molecules showed good water solubility and probable oral absorption. Molecules with a Log P value between 0 and 3 Log mol/L reflected good permeation and distribution behavior. Among the 52 selected iridoids, a total of 28 molecules showed good partition coefficient values. Molecules with Caco-2 cell permeability greater than −5.15 Log cm/s reflected a good probability of reaching the systemic circulation. Other than 10-Isovaleroyl-dihydropenstemide, Garjasmine, Ninpogenin, and Sotorasib, the rest of the molecules showed good Caco-2 cell permeation. The blood–brain barrier was permeated with Amphicoside, Garjasmine, Gentiopicroside, Laciniatoside II, Oleoside dimethyl ester, Picroside II, and Oleuropein. All molecules were noninhibitors of CYP1A2 and CYP2C19 microsomal enzymes. Only Sotorasib inhibited CYP2C9 and CYP2D6 microsomal enzymes. Acetylgeniposide, Buddlejoside A9, sylvestroside IV, Verminoside, and Sotorasib inhibited the CYP3A4 enzyme. The Lipinski rule reflects the permeability of a drug with a molecular weight of less than 500 g/mol, a Log P value of less than 5, and a maximum of 5 H-donor and 10 H-acceptor atoms. The Pfizer rule considers a molecule with LogP value greater than 3.0 and a TPSA less than 75 toxic. As per the GSK rule, a molecule with a molecular weight less than or equal to 400 Da and with a LogP value less than is four considered favorable. 10-Isovaleroyl-dihydropenstemide, 10-*O*-acetylgeniposide, 10-*O*-succinoylgeniposide, 7-hydroxy eucommiol, 8-epideoxyloganic acid, Asperuloside, Acetylbarlerin, Barlerin, brasoside, Deacetyl asperuloside, Euphroside, Garjasmine, Geniposidic acid, Gentiopicroside, Loganic acid, Mussaenoside, Ninpogenin, Oleoside dimethyl ester, Patrinalloside A, Pinnatoside, Plantarenaloside, and Polystachyn A followed the Lipinski rule. All molecules, including Sotorasib, followed thePfizer rule. 7-hydroxy eucommiol, brasoside, Euphroside, Garjasmine, Geniposidic acid, Gentiopicroside, Loganic acid, Mussaenoside, and Ninpogenin followed the GSK rule (Table S2). The toxicity profile of all the molecules reflected that Buddlejoside A9, Canteyloside, and Sotorasib were poor hERG blockers. Except for 6-*O*-alpha-d-galactopyranosylharpagoside, 6′-*O*-sinapoyl-geniposide, 6′-*O*-*trans*-*para*-coumaroylgeniposide, 8-*p*-coumaroylharpagide, Euphroside, Garjasmine, Kutkin, Laciniatoside I, Laciniatoside II, Mussaenoside, Picroside-III, Pinnatoside, Plantarenaloside, Shanzhiside methyl ester, and Specioside, the rest showed possible mild-to-moderate drug-induced liver injuries. Acetylgeniposide, Asperuloside, brasoside, Eurostoside, Garjasmine, Ninpogenin, Oleoside dimethyl ester, Oleuropein, Pinnatoside, Polystachyn A, sylvestroside III, and sylvestroside IV showed possible mild-to-moderate AMES toxicity. Only Amphicoside, Canteyloside, Garjasmine, Laciniatoside II, Ninpogenin, Picroside II, Polystachyn A, sylvestroside I, and sylvestroside III dimethyl acetal showed mild-to-moderate acute toxicity. Except 6-*O*-alpha-d-galactopyranosylharpagoside, 7-hydroxy eucommiol, Kutkin, Laciniatoside I, Loganic acid 6′-*O*-beta-d-glucoside, and Pinnatoside, rest molecules showed mild-to-moderate carcinogenicity (Table S3) [22].

## 3. Discussion

KRAS is a member of the rat sarcoma viral oncogene (RAS) family, which also includes two other isoforms in humans: HRAS and NRAS. The KRAS gene provides instructions for producing a protein called K-Ras, which is a crucial component of the RAS/MAPK pathway. The K-Ras protein functions as a GTPase, meaning it catalyzes the conversion of guanosine triphosphate (GTP) into guanosine diphosphate (GDP). This conversion allows the K-Ras protein to act as a molecular switch, turning signaling within the cell on or off. When K-Ras is activated by binding to GTP, it transmits signals downstream to the cell’s nucleus. However, when K-Ras hydrolyzes GTP into GDP, it becomes inactivated and cannot transmit signals. It is worth noting that the term “KRAS” is often specifically used to refer to the KRAS-4B isoform, which is the predominant isoform in cells because of the high levels of mRNA encoding KRAS-4B. This isoform plays a significant role in cancer development and progression. Understanding the mechanisms and dysregulation of KRAS and the RAS/MAPK pathway is crucial for developing targeted therapies for cancers associated with KRAS mutations. The RAS protein functions as a signaling GTPase, meaning it can switch between an active state when bound to GTP and an inactive state when bound to GDP. The activation of RAS involves the exchange of GDP for GTP, facilitated by guanine nucleotide exchange factors (GEFs). On the other hand, the inactivation of RAS involves the hydrolysis of GTP into GDP, which is facilitated by GTPase-activating proteins (GAPs). The activation of receptor tyrosine kinases (RTKs)-such as the epidermal growth factor receptor (EGFR) family, located on the plasma membrane—triggers the activation of KRAS. This activation leads to the activation of multiple downstream effector pathways, including the mitogen-activated protein kinase (MAPK) pathway and the phosphatidylinositol 3-kinase (PI3K) pathway. SOS Ras/Rac guanine nucleotide exchange factor 1 (SOS1) and protein tyrosine phosphatase non-receptor type 11 (PTPN11 or SHP2) are examples of proteins involved in the activation of KRAS downstream of RTKs. They promote the exchange of GDP for GTP, resulting in the activation of KRAS. When comparing the structures of KRAS in its GTP-bound and GDP-bound states, two regions called switch-I and switch-II have been identified. Mutations, such as the commonly found cysteine 12 mutation, can disrupt the guanine exchange cycle. These mutations lock KRAS in an inactive GDP-bound form, preventing it from switching to the active state and leading to the propagation of pro-tumorigenic signals. Understanding these mechanisms and the impact of KRAS mutations is crucial for developing targeted therapies aimed at inhibiting the aberrant signaling caused by mutated KRAS and its associated cancer-promoting effects. Sotorasib (AMG510) and Adagrasib (MRTX849) are examples of covalent inhibitors that have shown promise in clinical trials specifically targeting the KRAS-G12C mutation. Iridoids inhibit cancer cell growth by inducing cell cycle arrest or by regulating apoptosis-related signaling pathways. They can also suppress the expression and activity of matrix metalloproteinases (MMPs), which are enzymes involved in cancer cell migration and invasion. In this study, a total of 52 iridoids were selected and targeted the KRAS G12C (8AFB) receptor. However, in the discussion section, we only focused on the best-docked iridoids and standard Sotorasib. This work consists of different steps, including the selection and refinement of iridoid molecules; the identification and refinement of the KRASG12C receptor; identifying the best drug–receptor interaction using molecular docking studies; identifying the physical movements of atoms present within the receptor upon interaction with ligand molecules via MD simulation; identifying stable protein–ligand interactions at the atomic level via MM/PBSA analysis; and highlighting the electronic nature and electronic distribution of molecules via FMO and MEP analysis, followed by a pharmacokinetic–toxicity behavior analysis using ADMET profiling. All these parameters help identify the best molecule effective against KRAS G12C mutation. Among all the molecules, 6-*O*-*trans*-*p*-coumaroyl-8-*O*-acetylshanzhiside methyl ester and 6′-*O*-*trans*-*para*-coumaroyl geniposidic acid showed good docking interaction scores compared with standard Sotorasib. Sotorasib interacted with MET 72, ILE 100, GLN 99, ASP92, LYS 88, CYS 12, and ARG 68 via van der Waal interactions; GLU 62 via hydrogen bond interactions; and GLN 61 and TYR 96 via pi–pi interactions. CYS 12, TYR 96, and ARG 68 were the common amino acid residues between LXD and Sotorasib. 6-*O*-*trans*-*p*-coumaroyl-8-*O*-acetylshanzhiside methyl ester interacted with LYS 88, ASP 92, TYR 96, ASP 69, GLU 63, and ARG 68via hydrogen bond interactions; ALA 11, THR 58, ALA 59, GLN 61, THR 64by van der Waal interactions; MET 72via pi–pi interactions; and CYS 12via pi–sulfur interactions. ASP 92, TYR 96, ASP 69, ARG 68, CYS12, ALA 59, and GLN 61 were the common amino acid residues between co-crystallized ligands and 6-*O*-*trans*-*p*-coumaroyl-8-*O*-acetylshanzhiside methyl esters. 6′-*O*-*trans*-*para*-coumaroylgeniposidic acid interacted with ASP 69, THR 58, GLY10, ALA59, and LYS88 via hydrogen bond interactions; GLU 63, ARG 68, TYR 64, PHE78, GLN 61, ASP 92, and GLN 99via van der Waal interactions; and MET 72via pi-sulfur interactions. ASP 69, ALA 59, GLU 63, and ARG 68 shared common amino acid residues between LXD and 6′-*O*-*trans*-*para*-coumaroylgeniposidic acid. 6-*O*-*trans*-cinnamoyl secologanoside interacted with ARG 68, GLY10, THR 58, ALA 59, and LYS 88 via hydrogen bond interactions; CYS 12 and MET 72 via pi-sulfur interactions; and GLN 99, GLN 61, TYR 96, LYS 16, and ALA 11 via van der Waal interactions. ARG 68, ALA 59, CYS 12, GLN 99, and TYR 96 shared common amino acid residues between LXD and 6-*O*-*trans*-cinnamoyl-secologanoside. Loganic acid 6′-*O*-beta-d-glucoside interacted with TYR 96, ASP92, ASP69, GLY10, THR58, and ALA59 via hydrogen bond interactions; HIS95, GLU62, ALA11, GLN99, TYR64, GLU63, MET72, ARG68, GLN61, and CYS12via van der Waal interactions. TYR 96, ASP92, ASP69, GLU62, ARG68, GLN61, and CYS12 shared common amino acid residues between co-crystallized ligand and Loganic acid 6′-*O*-beta-d-glucoside. 10-*O*-succinoylgeniposide interacted with ARG 102, ARG 68, THR 58, GLY 10, ALA 59, TYR 96, ASP 92, and GLY60 via hydrogen bond interactions; MET 72and CYS 12 via pi-sulfur interactions; GLN99via pi-pi interactions; and LYS 16, TYR 64, GLU 62, HIS 95, and ALA 11 via van der Waal interactions. ARG 102, ARG 68, ALA59, TYR 96, ASP 92, GLY 60, GLN99, GLU 62, HIS 95, and CYS 12 shared common amino acids between LXD and 10-*O*-Succinoylgeniposide. Loganic acid interacted with TYR 96, GLY10, THR 58, ARG68, ALA 59 via hydrogen bond interactions; LYS 16, ILE 100, GLN99, VAL 9, GLU 63, VAL 103, ASP69, and GLY60 via van der Waal interactions; TYR64, and MET72 via pi-sulfur interactions. TYR 96, ARG 68, ALA 59, ILE 100, GLN99, GLU 63, VAL 103, ASP69, and GLY60 shared common amino acid residues between co-crystallized ligand and Loganic acid. Amphicoside interacted with LYS 88, ASP 92, HIS 95, THR 58, ASP69, ARG 102, TYR64, and GLU63 via hydrogen bond interactions; VAL 9 and GLN 99 via van der Waal interactions; MET 72via pi-sulfur interactions; and ARG 68 and TYR 96via pi-pi interactions. ASP 92, ASP69, ARG102, GLU63, GLN99, ARG 68, and TYR96 shared common amino acid residues between LXD and Amphicoside. These common amino acid residues between iridoids and co-crystallized ligands (LXDs) confirmed that all the iridoids perfectly docked within the 8AFB receptor’s active site. The 100ns MD simulation data showed that almost all molecules attained a static value in the RMSD, except for 6-*O*-*trans*-cinnamoyl secologanoside, which showed a sudden surge in values within the 50–80 ns range. The RMS fluctuations were not very significant in hampering the structural integrity. The radius of the gyration value is directly correlated with the structural stability. A high value means less stability and vice-versa. Lower-range values of the radius of gyration represent higher stability. SASA values were almost constant throughout the analysis, which confirmed that SASA values positively impacted the binding energy. Here, lower SASA values in the complexes showed higher stability. The SASA values of all the molecules attained a lower value near 90 nm^2^, which confirmed that they were highly stable complexes. Overall, the ligand–receptor complex showed good structural integrity upon interaction. All the parameters were within the limit and did not create any disturbances. The MM/PBSA data showed that, for 6-*O*-*trans*-*p*-coumaroyl-8-*O*-acetylshanzhiside methyl ester-8AFB complex, the van der Waal energy (−5.656 kJ/mol) was positively impacted in the interaction following the application of electrostatic energy (−2.468 kJ/mol) and SASA energy (−0.828 kJ/mol). The standard Sotorasib showed a binding energy of −3.551 kJ/mol. In all cases, polar salvation energy negatively impacted the binding energy. The FMO analysis data showed that the reactive nature and stability of the molecules were as follows: Sotorasib > 6-*O*-*trans*-*p*-coumaroyl-8-*O*-acetylshanzhiside methyl ester >6′-*O*-*trans*-*para*-coumaroyl geniposidic acid. In addition, the molecules were also characterized based on their reactivity, chemical hardness (η), electronegativity (μ), softness (ζ), and electrophilicity index (Ψ) [23]. Among the selected iridoids, 6-*O*-*trans*-*p*-coumaroyl-8-*O*-acetylshanzhiside methyl ester was referred to as a soft molecule, as well as by 6′-*O*-*trans*-*para*-coumaroyl geniposidic acid, because smaller energy gap systems are referred to as soft molecules. The chemical behaviors of 6-*O*-*trans*-*p*-coumaroyl-8-*O*-acetylshanzhiside methyl ester and 6′-*O*-*trans*-*para*-coumaroyl geniposidic acid were also identified using electronegativity factors. A greater electronegativity index value indicated higher chemical reactivity. Here, maximum electronegativity values of 5.72, 4.28, and 3.91 were observed in Sotorasib (standard), 6-*O*-*trans*-*p*-coumaroyl-8-*O*-acetylshanzhiside methyl ester, and 6′-*O*-*trans*-*para*-coumaroyl geniposidic acid, respectively. Thus, among the iridoids, 6-*O*-*trans*-*p*-coumaroyl-8-*O*-acetylshanzhiside methyl ester was the most likely chemically reactive molecule, followed by 6′-*O*-*trans*-*para*-coumaroyl geniposidic acid. The HOMO and LUMO orbitals of 6-*O*-*trans*-*p*-coumaroyl-8-*O*-acetylshanzhiside methyl ester were focused on the 4-hydroxy cinnamoyl groups. The HOMO and LUMO orbitals of 6′-*O*-*trans*-*para*-coumaroyl geniposidic acid were focused on the 4-hydroxy cinnamoyl groups. In the case of Sotorasib, HOMO and LUMO orbitals focused on 2-methyl piperizine-4-carbonyl and some portions of the pyrido-pyrimidine system, respectively. The MEP data showed the possible electrophilic and nucleophilic attack regions of the selected iridoids, which helps to understand possible interaction profiles [24]. The aqueous solubility of a drug molecule is an essential parameter in systemic absorption. If a Log S value ranges between −4 and 0.5 Log mol/L, it is considered to have good water solubility. All the best-docked iridoids showed good aqueous solubility, except Sotorasib. The partition coefficient is an important parameter of a drug molecule in permeating through biological membranes with a proper distribution coefficient. Molecules with a Log P value of 0–3 Log mol/L are considered to have the optimal value. Among the considered molecules, 6-*O*-*trans*-cinnamoyl-secologanoside, 6′-*O*-*trans*-*para*-coumaroylgeniposidic acid, 6-*O*-*trans*-*p*-coumaroyl-8-*O*-acetylshanzhiside methyl ester, and 10-*O*-succinoylgeniposide showed good partition coefficient values. Caco-2 cell (human colon adenocarcinoma cell line) permeability is associated with the in vivo permeation of drug molecules and reaches systemic circulation via passive diffusion, carrier-mediated uptake, and an active transport process. If the predicted value is greater than −5.15 Log cm/s, it represents excellent permeation. All the considered iridoids showed good Caco-2 permeability [25]. All the best-docked Iridoids were non inhibitors of P glycoprotein except standard Sotorasib. P glycoprotein inhibitors may show potential toxicity. Blood–brain barrier permeability is an important factor when a drug acts on the central nervous system. Of the selected molecules, only Amphicoside showed a slight inclination toward blood–brain barrier permeation. Most of the microsomal enzymes (CYP 1A2/2C9/2C19/2D6/3A4) are responsible for phase I biotransformation in the liver. All the best-docked molecules were noninhibitors of microsomal enzymes. Standard Sotorasib showed probable inhibitions of CYP2C9/2D6/3A4. The ADMET data showed that, among all the best-docked molecules, only Loganic acid followed the Lipinski, Pfizer, and GSK rules.All the best-docked molecules and the standard showed mild-to-moderate drug-induced liver injuries. Except for Loganic acid 6′-*O*-beta-d-glucoside, all the best-docked ligands showed probable carcinogenicity. Only Amphicoside showed very mild oral toxicity. No molecules showed AMES mutagenic behavior [26]

## 4. Materials and Methods

### 4.1. Molecular Docking Studies

#### 4.1.1. Selection and Preparation of the Receptor

Molecular docking study was used to predict ligand–target interactions by calculating the binding energy. In molecular docking study, ligand and target proteins interacted to create an environment that inhibited receptor activity via ligand molecules, and the output features reflected the possible activity of the ligand molecule. The interactive pose of the ligand molecules within the receptor reflected the structural features of the ligand upon interaction with the receptor. The output energy tabulation represented the highest to lowest docking conformers. In this study, the selected 52 iridoids were docked against the 8AFB receptor. The 8AFB receptor belongs to the oncoprotein class obtained from *Homo sapiens*, with *E. coli* as the expression system. The receptor contains 1 chain with 170 amino acids. (4~{S})-2-azanyl-4-[3-[6-[(2~{S})-2,4-dimethylpiperazin-1-yl]-4-(4-prop-2-enoylpiperazin-1-yl)pyridin-2-yl]-1,2,4-oxadiazol-5-yl]-4-methyl-6,7-dihydro-5~{H}-1-benzothiophene-3- carbonitrile (LXD) was present as a complexed ligand. A Ramachandran plot of 8AFB showed 92.6% residues within the most favored region (Figure S2). Before proceeding with the molecular docking studies, receptors were checked for missing residues using the PyMol software, followed by maintaining the ionization state of the amino acids present in the receptor using the H^++^ server. Then, the surrounding residues of the co-crystallized ligands were evaluated via Drug Discovery Studio. Themolecular docking study was performed using the AUTODOCK Vina software and visualized using the Drug Discovery Studio software [27].

#### 4.1.2. Preparation of Ligand Molecules

The structures of the selected 52 iridoids, co-crystallized ligand LXD, and standard Sotorasib were developed using the Avogadro software. The molecules were optimized using the MMFF94 force field and steepest descent methods. Then, all three-dimensional coordinates were added to the structure using the Open Babel software. Then, all the polar hydrogens and gasteiger charges were added to the molecules using AUTODOCK Vina and saved in the PDBQT format [28].

#### 4.1.3. Molecular Docking Parameters of the Molecules Interacting with the Receptors

Receptor and ligand molecules were charged with gasteiger charges and saved in the PDBQT format.The Lamarckian genetic algorithm was applied to seek the best binding site of ligands in respective proteins. Protein–ligand docking default parameters with grid box dimensions are listed below. Conformations were set as the output for the ligand–protein docking experiments. Finally, the docking results were visualized and analyzed using BIOVIA Discovery Studio Visualizer 4.5 and Ligplus. The grid box size of the 8AFB receptor was center_x = 18.479, center_y = −8.144, center_z = 22.453 and size_x = 24, size_y = 24, size_z = 24, with exhaustiveness = 8 [29].

### 4.2. MD Simulation Study

Molecular dynamic simulations help understand the structural atomic level dynamics of a ligand molecule interaction with a receptor. In this study, the GROMACS 20.1 software package was used to study the thermodynamic characteristics of the ligand–receptor complex. In this process, we separated the ligand molecule from the protein–ligand complex before beginning the simulation study, and then, we prepared GROMCAS-compatible .gro files with the help of the CHARMM 36 force field. Then, TIP3P water molecules, solvent molecules, sodium, and chloride ions were added to the processed files. The energies of the processed files were minimized using the steepest descent algorithm process with a 100 ns MD simulation run. The energy minimization process was processed in two distinct steps. In the initial step, the number of particles, volume, and temperature (NVT) remained constant, followed by choosing the number of particles, pressure, and temperature factors as constants. In this study, a total of 50,000 energy minimization steps were used. The trajectories of the MD simulation were studied using the GROMACS software package. The RMSD and RMSF of the protein–ligand complexes were computed using the gmx rms and gmx rmsf applications, respectively. The SASA (solvent-accessible surface area) and Rg (radius of gyration) were computed using the gmx sasa and gmx gyrate tools, respectively. Hydrogen bond formation was analyzed using the gmx hbond tool. The Qtgrace software was used to plot the graphical representation of the trajectories [30].

### 4.3. MM/PBSA: Calculation of Binding-Free Energy

To quantify the binding energy between the simulated iridoids and the 8AFB receptor, molecular mechanics Poisson–Boltzmann surface area (MM/PBSA) calculations were performed using the g_mmpbsa program. After the analysis, van der Waal energy, electrostatic energy, polar solvation energy, SASA energy, and binding energy were calculated [31].

### 4.4. FMO Analysis

The electric and optical parameters of the synthesized molecules in the frontier molecular orbital (FMO) theory were computed using B3LYP/6-31+G(d,p) basis sets. The charges of the molecules were zero, with one multiplicity. FM orbitals are the most important orbitals in a structure, and they are considered the lowest molecular orbitals (LUMOs) and the highest molecular orbitals (HOMOs). The outermost orbital occupied by electrons is called the HOMO, which can act as an electron donor [32]. The energy of the LUMO is the foremost vacant innermost orbital unoccupied by electrons, and it serves as an electron acceptor. The electron-donating capacity of a molecule is associated with the E_HOMO_, and the greater the HOMO energy (smaller negative value), the better the capacity to donate electrons [33]. The energy gap (ΔE) between the HOMO and LUMO, softness, electronegativity, chemical hardness, and electrophilicity index values were calculated. Here, we used the GAMESS software for FMO analysis, and WxMacMolPlt was used to visualize the data [34].

Chemical hardness: η = $\frac{I-A}{2}$; softness ζ = $\frac{1}{2\eta }$; electronegativity: μ = $-\frac{I+A}{2}$; electrophilicity index: Ψ = $\frac{\mu \;2}{2\eta }$, where A and I are electron affinity and ionization potential. A = −E_LUMO_ and I = −E_HOMO_.

### 4.5. MEP Investigation

The MEP (interaction energy at a certain zone of a structure) was involved in electrical charge distribution from the protons, nuclei, and electrons [35]. This electrostatic potential map showed attractive or repulsive forces observed with a fixed charge (a point positive charge, i.e., a proton) at various points in space that are equidistant from a molecular surface. This map also expressed electron-rich and electron-deficient regions. The probable nucleophilic (blue region) and electrophilic (red region) attack sites were established. The MEP was computed at the B3LYP-functional and 6–31+ G(d,p) level of theory for 6-*O*-*trans*-*p*-coumaroyl-8-*O*-acetylshanzhiside methyl ester, 10-*O*-succinoyl geniposide, Loganic acid 6′-*O*-beta-d-glucoside, 6-*O*-*trans*-cinnamoyl secologanoside, Loganic acid, 6-*O*-*trans* coumaroyl geniposidic acid, Amphicoside, and Sotorasib, which provides the valuable evidence about the possible nucleophilic and electrophilic sites present in the structure [36].

### 4.6. ADMET Calculation

The ADMET behaviors, including MW, TPSA, LogS, LogP, Caco-2 permeability, Pgp inhibitor, Pgp substrate, BBB penetration, CYP 1A2/2C19/2C9/3A4/2D6 inhibitor, the Lipinski rule, the Pfizer rule, hERG blocker, drug-induced liver injury, AMES toxicity, rat oral acute toxicity, carcinogenicity, respiratory toxicity, skin sensitivity, and eye irritation of all the selected iridoids were calculated using the ADMETlab 2.0 server (https://admetmesh.scbdd.com/ (accessed on 20 June 2023) [37].

## 5. Conclusions

The importance of iridoids has been recognized in recent years because of improvements in methods of extracting, storing, and isolating natural components. Mutated KRAS is responsible for pancreatic, lung, and colon cancers. Among KRAS mutations, G12 mutations are the most common, accounting for around 89% of cases. Within G12 mutations, G12D is the most prevalent (36%), followed by G12V (23%) and G12C (14%). G13 mutations make up about 9% of cases, and Q61 mutations are the least common at around 1%. Different cancer types exhibit varying frequencies of specific KRAS mutation subtypes. G12D plays a major role in pancreatic adenocarcinoma, while G12C is the most common subtype in non-small-cell lung cancer. Recently, Sotorasib and Adagrasib showed ground-breaking performance against the KRASG12C mutation. In this work, we established the potential of iridoids as KRASG12C inhibitors using molecular docking studies using 8AFB, MD simulation, MM/PBSA, FMO, and MEP analysis. There is enough evidence to say that natural anticancer molecules (Vincristine, Vinblastine, Paclitaxel, Docetaxel, etc.) do not conform with Lipinski, GSK, and other drug-likeness parameters, but they are still effective in controlling cancer progression. Thus, by keeping this information in mind, we can say that these computational data require both in vivo pharmacological and toxicological experiments to establish these molecules as anticancer agents. The main importance of this work is repurposing iridoids in the treatment of the KRASG12C mutation and its associated cancers. In conclusion, if we consider 6-*O*-*trans*-*p*-coumaroyl-8-*O*-acetylshanzhiside methyl ester, 10-*O*-succinoylgeniposide, Loganic acid 6′-*O*-beta-d-glucoside, 6-*O*-*trans*-cinnamoyl-secologanoside, Loganic acid, 6′-*O*-*trans*-*para*-coumaroyl geniposidic acid, and Amphicoside as KRASG12C inhibitors, they will be effective and logical weaponsin the treatment of cancer. Given the complicated nature of KRASG12C, to date, only two drug molecules (Sotorasib and Adagrasib) have been developed. We hope our study opens a new dimension in the treatment of KRASG12C using naturally developed iridoids.

## Figures and Tables

**Table 1:** Molecular docking interaction scores and interacting residues of iridoids, LXD, and Sotorasib.

Sr. No	Name of the Molecules	Docking Score (kcal/mol)	Interacting Amino Acid Residues
1.	6-*O*-alpha-d-galactopyranosylharpagoside	−7.8	CYS12 ^b^, LYS 88 ^b^, ASP 92 ^b^, GLY 60 ^b^, TYR 96 ^b^ THR 35 ^a^, GLN 61 ^c^, GLU 62 ^c^, ARG 68 ^c^, GLU 63 ^c^, TYR 64 ^c^, MET 72 ^d^, ALA 59 ^c^, ALA 11 ^a^
2.	6′-*O*-sinapoyl-geniposide	−9.0	GLU 63 ^b^, ARG 68 ^b^, GLN 61 ^b^, ALA 59 ^b^, PHE 78, TYR 64 ^a^, ASP 69 ^a^, GLN 99 ^b^, HIS 95 ^c^, ASP 92 ^c^, ALA 11 ^a^, HIS 94 ^a^, LYS 16 ^a^, THR 58 ^a^
3.	6-*O*-*trans*-cinnamoyl-secologanoside	−9.5	ARG 68 ^b^, GLY10 ^b^, THR 58 ^b^, ALA 59 ^b^, LYS 88, CYS 12 ^d^, MET 72 ^d^, GLN 99 ^a^, GLN 61, TYR 96 ^a^, LYS 16 ^a^, ASP 92 ^b^, ALA 11 ^a^
4.	6′-*O*-*trans*-*para*-coumaroylgeniposide	−9.0	TYR 96 ^b^, GLY 10 ^b^, ALA 59 ^b^, TYR 64 ^b^, HIS 95 ^b^, ASP 92 ^a^, CYS 12 ^b^, LYS 88 ^a^, ALA 11 ^a^,GLU 62 ^a^, GLN 61 ^a^, ARG 68 ^a^, THR 58 ^a^, GLN 99 ^a^, PHE 78 ^a^, MET 72 ^d^.
5.	6′-*O*-*trans*-*para*-coumaroylgeniposidic acid	−9.6	ASP 69 ^b^, THR 58 ^b^, GLY10 ^b^, ALA59 ^b^, LYS88 ^b^, GLU 63 ^a^, ARG 68 ^a^, TYR 64 ^a^, PHE78 ^a^, GLN 61 ^a^, ASP 92 ^a^, MET 72 ^d^, GLN 99 ^a^.
6.	6-*O*-*trans*-*p*-coumaroyl-8-*O*-acetylshanzhiside methyl ester	−9.9	LYS 88 ^b^, ASP 92 ^b^, TYR 96 ^b^, ASP 69 ^b^, GLU 63 ^b^, ARG 68 ^b^, ALA 11 ^a^, CYS 12 ^d^, THR 58 ^a^, ALA 59 ^a^, GLN 61 ^a^, THR 64 ^a^, MET 72 ^c^.
7.	7-hydroxy eucommiol	−5.8	ARG 68 ^b^, GLU 63 ^b^, TYR 96 ^b^, TYR 64 ^a^, GLU 62 ^a^, HIS 95 ^a^, GLN 99 ^a^, MET 72 ^d^, THR 58 ^c^, VAL9 ^c^.
8.	8-epideoxyloganic acid	−8.4	TYR 96 ^b^, ALA 59 ^b^, CYS 12 ^d^, GLU 62 ^c^, GLN 61 ^c^, GLU 63 ^a^, TYR 64 ^a^, ARG 68 ^a^, MET 72 ^d^, PHE 78 ^c^, GLN99 ^a^.
9.	8-*p*-coumaroylharpagide	−8.1	LYS 88 ^b^, CYS 12 ^b^, ASP 92 ^b^, GLY 60 ^b^, TYR 96 ^b^, ALA 11 ^a^, GLU 62 ^a^, ALA 59 ^a^, GLN 61 ^c^, VAL 9 ^c^.
10.	10-Isovaleroyl-dihydropenstemide	−8.3	TYR 96 ^b^, ARG 102 ^b^, ASP 69 ^b^, LYS 88 ^a^, LYS 16 ^a^, GLY 10 ^a^, ALA 59 ^a^, THR 58 ^c^, VAL 9 ^c^, MET 72 ^d^, TYR 64 ^a^, GLN 99 ^a^, ASP 92 ^a^.
11.	10-*O*-acetylgeniposide	−8.7	ARG 102 ^b^, ARG 68 ^b^, TYR 96 ^b^, GLN 61 ^b^, ALA59 ^b^, HIS 95 ^a^, GLU 62 ^a^, TYR 64 ^a^, MET 72 ^d^, GLN 99 ^c^, GLY 10 ^a^, THR 58 ^c^, LYS 16 ^a^.
12.	10-*O*-succinoylgeniposide	−9.4	ARG 102 ^b^, ARG 68 ^b^, THR 58 ^b^, GLY 10 ^b^, ALA 59 ^b^,TYR 96 ^b^, ASP 92 ^b^, GLY60 ^b^, MET 72 ^d^, GLN99 ^c^, LYS 16 ^a^, TYR 64 ^a^, GLU 62 ^a^, HIS 95 ^a^, CYS 12 ^d^, ALA 11 ^a^.
13.	Acetylgeniposide	−6.6	LYS 88 ^b^, TYR 96 ^b^, TYR 64 ^b^, ARG 68 ^b^, CYS 12 ^d^, ALA 11 ^a^, GLY60 ^a^, ALA59 ^a^, THR 58 ^a^, HIS 95 ^a^, GLN 99 ^a^, GLU 62 ^c^ MET 72 ^d^, GLY 10 ^a^.
14.	Acetylbarlerin	−7.7	GLU 63 ^b^, GLN 61 ^b^, GLU 62 ^b^, THR 96 ^b^, CYS 12 ^b^, THR 58 ^b^, ARG 68 ^b^, VAL9 ^a^, ASP 92 ^a^, ALA 11 ^a^, MET 72 ^d^.
15.	Amphicoside	−9.2	LYS 88 ^b^, ASP 92 ^b^, HIS 95 ^b^, THR 58 ^b^, ASP69 ^b^, ARG 102 ^b^, TYR64 ^b^, GLU63 ^b^, VAL 9 ^a^, GLN 99 ^a^, MET 72 ^d^, ARG 68 ^c^, TYR 96 ^c^.
16.	Asperuloside	−8.9	ARG 68 ^b^, ALA59 ^b^, TYR96 ^b^, TYR 64 ^a^, GLN99 ^b^, VAL9 ^b^, GLN61 ^a^, GLU62 ^a^, HIS 95 ^c^, MET 72 ^d^.
17.	Barlerin	−8.4	ALA 59 ^b^, GLU 62 ^b^, ARG 68 ^b^, ARG 102 ^b^, TYR64 ^b^, HIS 95 ^b^, ASP 92 ^b^, TYR 96 ^b^, GLN 61 ^a^, GLY 60 ^a^, GLY 10 ^a^, THR 58 ^a^, ASP 69 ^c^, MET 72 ^d^, GLN99 ^c^.
18.	Brasoside	−8.6	ALA 59 ^b^, GLU 62 ^b^, ARG 68 ^b^, ARG 102 ^b^, TYR 64 ^b^, HIS 95 ^b^, ASP 92 ^b^, TYR 96 ^b^, GLN 61 ^a^, GLY 60 ^a^, GLY 10 ^a^, THR58 ^c^, ASP69 ^c^, MET 72 ^d^, GLN 99 ^c^.
19.	Buddlejoside A9	−7.5	CYS 12 ^b^, TYR 96 ^b^, GLY 60 ^b^, PRO34 ^a^, ALA 11 ^a^, GLU 62 ^a^, GLY 10 ^c^, TYR 64 ^a^, THR 58 ^a^, ARG 68 ^a^, ASP 69 ^c^, GLN 99 ^a^, ALA 59 ^a^, MET 72 ^d^, HIS 95 ^c^.
20.	Cantleyoside	−8.1	GLY 60 ^b^, TYR 96 ^b^, ARG 102 ^b^, ASP 69 ^b^, THR 58 ^b^, GLY 10 ^b^, PRO 34 ^b^, THR 35 ^a^, CYS 12 ^a^, TYR 64 ^a^, GLN99 ^c^, ARG 68 ^c^, MET 72 ^d^, LYS 16 ^c^, GLU 62 ^a^, ALA 59 ^a^.
21.	Deacetyl asperuloside	−8.6	TYR 96 ^b^, ALA 59 ^b^, ARG 68 ^b^, GLU 62 ^a^, GLU 63 ^a^, GLN 99 ^c^, MET 72 ^d^, CYS 12 ^d^.
22.	Euphroside	−8.6	GLY 10 ^b^, THR 58 ^b^, ASP64 ^b^, ARG 68 ^b^, ALA59 ^b^, TYR 96 ^b^, VAL9 ^a^, GLN 99 ^a^, MET 72 ^d^, TYR 64 ^c^, GLU 62 ^c^, GLY 60 ^c^, GLN 61 ^c^.
23.	Eurostoside	−8.6	ALA 59 ^b^, ARG 68 ^b^, THR58 ^b^, GLY 10 ^b^, GLY 60 ^a^, GLU62 ^a^, TYR96 ^c^, HIS 95 ^c^, TYR64 ^a^, GLN99 ^c^, MET72 ^d^, LYS 16 ^a^, VAL 9 ^a^, GLN61 ^c^, CYS 12 ^d^.
24.	Garjasmine	−7.2	TYR 64 ^b^, ARG 68 ^b^, GLU 62 ^a^, TYR 96 ^a^, GLN99 ^a^, PHE 78 ^a^, MET 72 ^d^, VAL 9 ^c^, GLN61 ^c^, GLU 63 ^c^.
25.	Geniposidic Acid	−8.9	CYS 12 ^b^, ALA 59 ^b^, THR58 ^b^, ARG68 ^b^, TYR64 ^b^, ARG 102 ^b^, HIS 95 ^b^, ASP92 ^b^, TYR96 ^b^, GLY 10 ^b^, MET 72 ^d^, ASP69 ^c^, GLN 99 ^a^, GLU 62 ^a^, LYS 16 ^a^.
26.	Gentiopicroside	−8.9	TYR 96 ^b^, GLY 10 ^b^, THR 58 ^b^, ARG 68 ^b^, ALA 59 ^b^, LYS16 ^a^, VAL9 ^a^, GLN99 ^c^, ILE100 ^c^, ASP69 ^a^, MET72 ^d^, TYR 64 ^a^, GLU63 ^c^, CYS12 ^d^.
27.	Isojaslanceoside B	−8.6	LYS 88 ^b^, CYS12 ^b^, ARG 68 ^b^, GLN61 ^b^, ASP69 ^b^, GLU63 ^b^, TYR64 ^b^, HIS 95 ^b^, ASP92 ^b^, GLU 62 ^a^, ALA59 ^a^, ARG102 ^a^, GLN99 ^a^, MET72 ^d^, TYR 96 ^c^.
28.	Kutkin	−1.8	SER 17 ^b^, ILE 36 ^b^, THR58 ^b^, MG202 ^b^, GDP 201 ^b^, ASP57 ^a^, ALA59 ^a^.
29.	Laciniatoside I	−8.6	ASP 92 ^b^, CYS12 ^b^, TYR 96 ^b^, GLU62 ^b^, ALA11 ^a^, GLN99 ^a^, ARG 68 ^a^, ILE100 ^c^, VAL103 ^c^, MET 72 ^d^, GLN 61 ^c^, VAL9 ^a^, GLU 63 ^c^, HIS 95 ^a^.
30.	LaciniatosideII	−8.7	ASP92 ^b^, HIS95 ^b^, TYR64 ^b^, ASP69 ^b^, GLU63 ^b^, CYS12 ^a^, GLU62 ^a^, GLY10 ^a^, ALA59 ^a^, ARG68 ^c^, LYS16 ^a^, MET 72 ^d^, VAL103 ^a^, GLN99 ^a^, ARG 102 ^c^.
31.	Loganic acid	−9.4	TYR 96 ^b^, GLY10 ^b^, THR 58 ^b^, ARG68 ^b^, ALA 59 ^b^, LYS 16 ^a^, ILE 100 ^a^, GLN99 ^a^, VAL 9 ^a^, TYR64 ^c^, MET72 ^c^, GLU 63 ^a^, VAL 103 ^a^, ASP69 ^a^, GLY60 ^a^.
32.	Loganic acid 6′-*O*-beta-d-glucoside	−9.5	TYR 96 ^b^, ASP92 ^b^, ASP69 ^b^, GLY10 ^b^, THR58 ^b^, ALA59 ^b^, HIS95 ^a^, GLU62 ^a^, ALA11 ^a^, GLN99 ^a^, TYR64 ^a^, GLU63 ^a^, MET72 ^a^, ARG68 ^a^, GLN61 ^a^, CYS12 ^a^.
33.	Minecoside	−8.9	TYR 96 ^b^, ARG102 ^b^, ARG68 ^b^, GLN99 ^a^, TYR64 ^a^, GLN61 ^a^, GLU62 ^c^, HIS95 ^c^, ASP92 ^c^, THR 58 ^a^, ALA59 ^a^, VAL 9 ^a^, MET 72 ^a^, GLU 63 ^a^, ILE 100 ^a^.
34.	Mussaenoside	−8.2	TYR 96 ^a^, TYR64 ^a^, HIS 95 ^a^, ARG 102 ^b^, ASP 92 ^a^, ARG 68 ^b^, GLY 60 ^b^, ALA 59 ^a^, GLN61 ^a^, VAL 103 ^a^, GLN99 ^a^, MET 72 ^c^, VAL 9 ^c^, GLU 62 ^a^.
35.	Ninpogenin	−6.0	GLN61 ^a^, GLU63 ^b^, ARG68 ^b^, ASP92 ^a^, HIS95 ^b^, TYR64 ^a^, GLU62 ^a^, MET 72 ^c^, GLN 99 ^a^, TYR 96 ^a^.
36.	Nuezhenelenoliciside	−8.7	ASP 92 ^b^, GLU 63 ^b^, ASP69 ^b^, ARG69 ^b^, ALA 59 ^b^, GLY60 ^b^, HIS 95 ^b^, TYR 64 ^b^, LYS 88 ^a^, TYR 96 ^a^, MET 72 ^c^, GLY 10 ^a^, VAL 9 ^a^, THR 58 ^a^, LYS 16 ^a^, GLN 61 ^a^, GLU 62 ^a^.
37.	Nuezhenide	−8.9	GLU62 ^b^, LYS 88 ^b^, CYS12 ^b^, TYR96 ^b^, GLY10 ^b^, ARG 68 ^b^, HIS 95 ^a^, ASP 92 ^a^, ALA 11 ^a^, LYS 16 ^a^, ALA59 ^a^, VAL9 ^a^, GLN 99 ^a^, ILE 100 ^a^, VAL 103 ^a^, MET 72 ^c^.
38.	Oleoside dimethyl ester	−7.1	GLU 62 ^b^, LYS 88 ^b^, ASP 92 ^b^, HIS 95 ^b^, ARG 68 ^b^, GLY 60 ^b^, CYS 12 ^a^, ALA 11 ^c^, TYR 96 ^a^, TYR 64 ^a^, MET 72 ^a^, ALA 59 ^a^, GLN 99 ^a^, GLU 63 ^a^, VAL 9 ^a^, VAL 103 ^a^, GLN 61 ^a^, THR 35 ^a^, PRO 34 ^a^.
39.	Oleuropein	−8.4	LYS 88 ^b^, ASP 92 ^b^, HIS 95 ^b^, TYR 64 ^b^, ARG 68 ^b^, TYR 96 ^c^, ALA 11 ^a^, VAL9 ^a^, GLN 99 ^a^, VAL103 ^c^, ILE 100 ^a^, ARG 102 ^a^, ASP 69 ^a^, ALA 59 ^a^, GLU 62 ^a^.
40.	Patrinalloside A	−8.9	GLU 62 ^b^, LYS 88 ^b^, ASP 92 ^b^, TYR 96 ^b^, THR 58 ^b^, GLY 10 ^b^, VAL 9 ^c^, GLN99 ^a^, ILE 100 ^c^, VAL 103 ^c^, MET 72 ^c^, LYS 16 ^a^, ALA 59 ^a^, HIS 95 ^a^.
41.	Picroside-II	−9.0	CYS 12, ASP 92, GLY 10, TYR 96, ARG 102, ARG 68; by H- bond interactions, ALA 11, GLN 61, GLU 62, GLU 63, TYR 64, MET 72, ALA 59, VAL 9, THR 58 by hydrophobic interactions.
42.	Picroside-III	−8.9	LYS 88 ^b^, HIS 95 ^b^, GLU 62 ^b^, TYR 64 ^b^, GLN99 ^b^, THR58 ^b^, GLY 10 ^b^, TYR 96 ^b^, ASP92 ^b^, VAL 103 ^a^, GLU 63 ^a^, ARG 68 ^a^, ASP 69 ^a^, VAL 9 ^a^, GLN 61 ^a^, ALA 11 ^a^, CYS 12 ^c^.
43.	Pinnatoside	−7.7	ALA 59 ^b^, THR 58 ^b^, ARG 68 ^b^, GLU 63, GLN 61 ^b^, GLU 62, LYS 16, TYR 96, GLN 99 ^b^,ARG 102 ^b^, ILE 100 ^a^, VAL 103 ^a^, MET 72 ^a^.
44.	Plantarenaloside	−8.6	TYR 96 ^c^, HIS 95 ^b^, ASP 92 ^b^, TYR 64 ^b^, ARG 68 ^b^, GLY 60 ^b^, ALA 59 ^b^, GLU 62 ^a^, GLN 99 ^a^, MET 72 ^c^, VAL 9 ^c^, THR 58 ^a^, GLN 61 ^a^.
45.	Polystachyn A	−8.5	ARG 68 ^b^, TYR 96 ^b^, GLN 61 ^a^, ALA59 ^a^, TYR 64 ^c^, GLU 62 ^a^, MET 72 ^c^, VAL 9 ^a^, GLU 63 ^a^, ASP 69 ^a^, GLN 99 ^a^, ARG 102 ^a^, VAL 103 ^a^, PHE 78 ^a^.
46.	Shanzhiside methyl ester	−8.4	GLU 62 ^b^, HIS 95 ^b^, ASP 92 ^b^, TYR 64 ^b^, ARG 68 ^b^, GLU 63 ^b^, TYR 96 ^b^, VAL 9 ^c^, GLN 99 ^a^, MET 72 ^c^, ILE 100 ^c^, ALA 11 ^a^, CYS 12 ^a^.
47.	Specioside	−8.6	LYS 88 ^b^, ASP92 ^b^, ARG 68 ^b^, GLU 63 ^b^, TYR 96 ^b^, TYR 64 ^a^, HIS 95 ^a^, VAL 9 ^a^, VAL103 ^c^, MET 72 ^c^, ILE 100 ^c^, PHE 78 ^a^, ALA 59 ^a^, CYS 12 ^a^, GLU 62 ^a^.
48.	Sylvestroside I	−8.0	GLU 63 ^b^, ASP 92 ^b^, HIS 95 ^b^, HIS 94 ^b^, GLU 98 ^b^, ARG 68 ^b^, GLN 61 ^a^, TYR 64 ^a^, GLU 62 ^a^, TYR 96 ^a^, VAL 9 ^c^.
49.	Sylvestroside III	−8.6	LYS 88 ^b^, ASP92 ^b^, TYR 64 ^b^, ASP 69 ^b^, HIS 95 ^b^, GLN 99 ^b^, ARG 102 ^b^, TYR 96 ^b^, ARG 68 ^b^, PHE 78 ^a^, MET 72 ^a^, VAL 103 ^a^, ILE 100 ^a^, GLU62 ^a^, GLU 63 ^a^, ALA 59 ^a^, GLN 61 ^a^.
50.	Sylvestroside III dimethyl acetal	−8.3	LYS 88 ^b^, CYS 12 ^b^, THR 58 ^b^, ASP 69 ^b^, TYR 64 ^b^, GLU 63 ^b^, TYR 96 ^b^, ALA 11 ^b^, ALA 59 ^b^, VAL 9 ^a^, ILE 100 ^a^, VAL 103 ^a^, HIS 95 ^a^, GLN 61 ^a^, GLU 62 ^a^.
51.	Sylvestroside IV	−8.8	CYS 12 ^b^, ASP 92 ^b^, TYR 96 ^b^, ARG 68 ^b^, ARG 102 ^b^, HIS 95 ^a^, GLY 10 ^a^, ALA 59 ^a^, THR 58 ^a^, LYS 16 ^a^, VAL 9 ^c^, MET 72 ^c^, GLU 63 ^a^, ASP 69 ^a^, GLN 99 ^a^, VAL 103 ^a^.
52.	Verminoside	−8.9	LYS 88 ^b^, ASP92 ^b^, HIS 95 ^b^, ARG 102 ^b^, ASP69 ^b^, TYR 64 ^b^, ALA 11 ^c^, CYS 12 ^d^, GLN 61 ^a^, GLU 62 ^a^, ALA 59 ^a^, ARG 68 ^a^, THR 58 ^a^, VAL 9 ^c^, MET 72 ^a^, GLN 99 ^a^, PHE 78 ^a^, ILE 100 ^a^, VAL 103 ^a^, TYR 96 ^a^.
53.	Sotorasib	−9.1	MET 72 ^a^, ILE 100 ^a^, GLN 99 ^a^, ASP92 ^a^, LYS 88 ^a^, GLU 62 ^b^, CYS 12 ^a^, GLN 61 ^c^, TYR 96 ^c^, ARG 68 ^a^.
54.	LXD	−11.7	ASP 92 ^b^, HIS 95 ^b^, TYR 64 ^b^, ASP 69 ^b^, GLU 63 ^b^, CYS 12 ^a^, GLU 62 ^a^, ALA 11 ^a^, LYS 88 ^a^, TYR 96 ^a^, VAL 9 ^c^, GLN 99 ^a^, VAL 103 ^c^, MET 72 ^d^, ARG 68 ^c^.

^a^ Van der Waal interaction; ^b^ hydrogen bonding; ^c^ pi-pi stacking ^d^ pi–sulfur interaction.

**Table 2:** MM/PBSA analysis data.

Sr. No.	Name of the Molecules	Van der Waal Energy (kJ/mol)	Electrostatic Energy (kJ/mol)	Polar Solvation Energy (kJ/mol)	SASA Energy (kJ/mol)	Binding Energy (kJ/mol)
1.	6-*O*-*trans*-*p*-coumaroyl-8-*O*-acetylshanzhiside methyl ester	−5.656	−2.468	1.643	−0.828	−7.309
2.	10-*O*-succinoylgeniposide	−16.228	−6.363	24.886	−2.391	−0.096
3.	Loganic acid 6′-*O*-beta-d-glucoside	−21.887	−10.770	33.348	−2.841	−2.150
4.	6-*O*-*trans*-cinnamoyl-secologanoside	−9.640	−11.466	27.814	−1.737	−4.971
5.	Loganic acid	−15.230	−6.953	25.109	−2.009	−0.917
6.	6′-*O*-*trans*-*para*-coumaroyl geniposidic Acid	−25.501	−5.342	23.845	−3.154	−6.153
7.	Amphicoside	−11.486	−3.441	12.217	−1.416	−4.216
8.	Sotorasib	−10.429	−1.621	9.543	−1.044	−3.551

**Table 3:** FMO analysis data.

Sr. No.	Name of the Molecules	E_HOMO_ (eV)	E_LUMO_ (eV)	ΔE gap (eV)	I	A	η	ζ	μ	Ψ
1.	6-*O*-*trans*-*p*-coumaroyl-8-*O*-acetylshanzhiside Methyl Ester	−6.44	−2.14	4.3	6.44	2.14	2.15	0.23	4.29	4.28
2.	10-*O*-succinoylgeniposide	−6.63	−1.06	5.57	6.63	1.06	2.78	0.17	3.85	2.66
3.	Loganic acid 6′-*O*-beta-d-glucoside	−6.83	−1.41	5.42	6.83	1.41	2.71	0.18	4.12	3.13
4.	6-*O*-*trans*-cinnamoyl-secologanoside	−6.55	−1.57	4.98	6.55	1.57	2.49	0.20	4.06	3.30
5.	Loganic acid	−6.80	−1.25	5.55	6.80	1.25	2.77	0.18	4.02	2.91
6.	6′-*O*-*trans*-*para*-coumaroyl geniposidic acid	−6.34	−1.95	4.39	6.34	1.95	2.19	0.22	4.14	3.91
7.	Amphicoside	−6.28	−1.49	4.79	6.28	1.49	2.39	0.20	3.88	3.14
8.	Sotorasib	−6.34	−2.74	3.6	6.34	2.74	1.8	0.27	4.54	5.72

## Data Availability

All the Supporting Materials can be found on the MDPI journal website.

## Supplementary Materials

The following supporting information can be downloaded at https://www.mdpi.com/article/10.3390/molecules28135050/s1: Figure S1: Interacting residues of complexed ligand LXD with 8AFB. Figure S2: Ramachandran plot of 8AFB. Table S1: List of iridoids and Sotorasib with their SMILE notifications.Table S2: Pharmacokinetic behavior of iridoids and Sotorasib. Table S3: Toxicity profile of iridoids and Sotorasib.

## Sample Availability

Samples of the compounds are not available from the authors.
