# Supplementary material for: Establishing the Role of Iridoids as Potential Kirsten Rat Sarcoma Viral Oncogene Homolog G12C Inhibitors Using Molecular Docking; Molecular Docking Simulation; Molecular Mechanics Poisson–Boltzmann Surface Area; Frontier Molecular Orbital Theory; Molecular Electrostatic Potential; and Absorption, Distribution, Metabolism, Excretion, and Toxicity Analysis
Source: Molecules. 2023 Jun 28;28(13):5050. doi: 10.3390/molecules28135050 (PMC10343556; doi:10.3390/molecules28135050)
Supplement: Supplementary file 1 [file molecules-28-05050-s001.zip › Table S1.pdf]

Table S1. List of Iridoids and Sotorasib with their SMILE Notifications

Sr.No	Name of the Molecules	Smile Notation
1.	6-O-alpha-D-galactopyranosylharpagoside	C[C@]1(C[C@@H]([C@]2([C@H]1[C@@H](OC=C2)O[C@H]1[C@@H]([C@@H]([C@@H]([C@@H](O1)CO)O)O)O)O)O[C@H]1[C@@H]([C@@H]([C@@H]([C@@H](O1)CO)O)O)O)OC(=O)/C=C/c1ccccc1
2.	6'-O-sinapoyl-geniposide	COc1cc(cc(OC)c1O)/C=C/C(=O)OC[C@H]1O[C@@H](O[C@H]2OC=C([C@@H]3CC[C@@H](CO)[C@@H]23)C(=O)OC)[C@@H](O)[C@@H](O)[C@H]1O
3.	6-O-trans-cinnamoyl-secologanoside	O=C(O)C1=CO[C@@H](O[C@H]2O[C@@H](COC(=O)/C=C\c3ccccc3)[C@@H](O)[C@@H](O)[C@H]2O)[C@@H](C=C)[C@H]1CC(=O)O
4.	6'-O-trans-para-coumaroylgeniposide	Oc1ccc(cc1)/C=C/C(=O)OC[C@H]1O[C@@H](O[C@H]2OC=C([C@@H]3CC[C@@H](CO)[C@@H]23)C(=O)OC)[C@@H](O)[C@@H](O)[C@H]1O
5.	6'-O-trans-para-coumaroylgeniposidic Acid	Oc1ccc(cc1)/C=C/C(=O)OC[C@H]1O[C@@H](O[C@H]2OC=C([C@@H]3CC[C@@H](CO)[C@@H]23)C(=O)O)[C@@H](O)[C@@H](O)[C@H]1O
6.	6-O-trans-p-coumaroyl-8-O-acetylshanzhiside methyl ester	C[C@@]1(C[C@H]([C@H]2[C@@H]1[C@@H](OC=C2C(=O)OC)O[C@H]1[C@@H]([C@H]([C@@H]([C@H](O1)CO)O)O)O)OC(=O)/C=C/c1ccc(cc1)O)O
7.	7-hydroxy eucommiol	OCC1=C(CO)[C@@H](CCO)[C@@H](O)[C@H]1O
8.	8-epideoxyloganic acid	C[C@@H]1CC[C@H]2[C@@H]1[C@@H](OC=C2C(=O)O)O[C@H]1[C@@H]([C@H]([C@@H]([C@H](O1)CO)O)O)O
9.	8-p-coumaroylharpagide	C[C@]1(C[C@@H]([C@]2([C@H]1[C@@H](OC=C2)O[C@H]1[C@@H]([C@@H]([C@@H]([C@@H](O1)CO)O)O)O)O)O)OC(=O)/C=C/c1ccc(cc1)O
10.	10-isovaleroyl-dihydropenstemide	CC(C)CC(=O)OC[C@H]1CC[C@H]2C(=CC[C@@H](OC(=O)CC(C)C)[C@H]12)CO[C@H]1O[C@@H](CO)[C@@H](O)[C@@H](O)[C@H]1O
11.	10-O-acetylgeniposide	CC(=O)OCC1=CC[C@H]2[C@@H]1[C@@H](OC=C2C(=O)OC)O[C@H]1[C@@H]([C@H]([C@@H]([C@H](O1)CO)O)O)O
12.	10-O-succinoylgeniposide	COC(=O)C1=CO[C@H]([C@H]2[C@@H]1CC=C2COC(=O)CCC(=O)O)O[C@H]1[C@@H]([C@H]([C@@H]([C@H](O1)CO)O)O)O
13.	Acetylgeniposide	CC(=O)OC[C@@H]1[C@H]([C@@H]([C@H]([C@@H](O1)O[C@H]1[C@H]2[C@H](CC=C2COC(=O)C)C(=CO1)C(=O)OC)OC(=O)C)OC(=O)C)OC(=O)C
14.	Acetylbarlerin	CC(=O)O[C@H]1C[C@]([C@H]2[C@@H]1C(=CO[C@@H]2O[C@H]1[C@@H]([C@H]([C@@H]([C@H](O1)CO)O)O)O)C(=O)OC)(C)OC(=O)C
15.	Amphicoside	O[C@H]1[C@@H](O)[C@@H](O)[C@@H](O[C@H]2OC=C(OC(=O)c3ccc(O)c(OC)c3)[C@@H]3C[C@@H]4O[C@]4(CO)[C@@H]23)O[C@H]1OC
16.	Asperuloside	O[C@H]1[C@@H](O)[C@@H](O)[C@@H](O[C@H]2OC=C3C(=O)O[C@H]4C[C@@H](COC(=O)C)[C@@H]2[C@@H]34)O[C@H]1OC
17.	Barlerin	CC(=O)O[C@]1(C[C@H]([C@H]2[C@@H]1[C@@H](OC=C2C(=O)OC)O[C@H]1[C@@H]([C@H]([C@@H]([C@H](O1)CO)O)O)O)O)C
18.	Brasoside	C[C@H]1C[C@H]2[C@H]3[C@@H]1[C@@H](OC=C3C(=O)O2)O[C@H]1[C@@H]([C@@H]([C@@H]([C@@H](O1)OC)O)O)O
19.	Buddlejoside A9	CC(=O)O[C@H]1[C@@H](O)[C@@H](O/C=C/c2ccc(OC)c(OC)c2)[C@@H](C)O[C@H]1C[C@H]1[C@H]2C=CO[C@@

		H](O[C@H]3O[C@@H](CO)[C@@H](O)[C@@H](O)[C@H]3O)[C@@H]2[C@@]2(C)O[C@@H]12
20.	Cantleyoside	C[C@H]1[C@@H](C[C@H]2[C@H]1[C@@H](OC=C2C(=O)OC)O[C@H]1[C@@H]([C@@H]([C@@H]([C@@H](O1)CO)O)O)O)OC(=O)C1=CO[C@@H]([C@@H]([C@H]1CC=O)C=C)O[C@H]1[C@@H]([C@@H]([C@@H]([C@@H](O1)CO)O)O)O
21.	Deacetyl asperuloside	O[C@H]1[C@@H](O)[C@@H](O)[C@@H](O[C@H]2OC=C3C(=O)O[C@H]4C[C@@H](CO)[C@@H]2[C@@H]34)O[C@H]1OC
22.	Euphroside	C[C@@]1(CC[C@]2([C@@H]1[C@@H](OC=C2C=O)O[C@H]1[C@@H]([C@H]([C@@H]([C@H](O1)CO)O)O)O)O)O
23.	Eurostoside	Oc1ccc(cc1)/C=C/C(=O)OCC1=C[C@@H](O)[C@H]2C=CO[C@@H](O[C@H]3O[C@@H](CO)[C@@H](O)[C@@H](O)[C@H]3O)[C@H]12
24.	Garjasmine	O=C(OC)C1=CO[C@H]2OC[C@@]3(O)C=C[C@@H]1[C@@H]23
25.	Geniposidic Acid	C1C=C([C@@H]2[C@H]1C(=CO[C@H]2O[C@H]1[C@@H]([C@H]([C@@H]([C@H](O1)CO)O)O)O)C(=O)O)CO
26.	Gentiopicroside	C=C[C@H]1[C@@H](OC=C2C1=CCOC2=O)O[C@H]1[C@@H]([C@H]([C@@H]([C@H](O1)CO)O)O)O
27.	Isojaslanceoside B	O[C@H]1[C@@H](O)[C@@H](O)[C@@H](O[C@H]2OC=C(C(=O)C)[C@@H](CCC(=O)O)[C@H]2CCOC(=O)/C=C\c2ccc(O)cc2)O[C@H]1OC
28.	Kutkin	COc1c(ccc(c1)C(=O)O[C@H]1[C@@H]([C@@H]([C@@H]([C@@H](O1)CO)O)O)O)OC(=O)/C=C/c1ccccc1.O.O
29.	Laciniatoside I	O[C@H]1[C@@H](O)[C@@H](O)[C@@H](CO)O[C@H]1O[C@H]1C[C@@H](CC=O)[C@@H](CO1)C(=O)O[C@H]1C[C@H]2[C@@H](CO[C@@H](O)[C@H]2C(=O)OC)[C@H]1C
30.	Laciniatoside II	CC1C(CC2C1COC(=O)C2)OC(=O)C3=COC(C(C3CC(O)O)C=C)OC4C(C(C(C(O4)CO)O)O)O
31.	Loganic acid	C[C@H]1[C@H](C[C@H]2[C@@H]1[C@@H](OC=C2C(=O)O)O[C@H]1[C@@H]([C@H]([C@@H]([C@H](O1)CO)O)O)O)O
32.	Loganic acid 6'-O-beta-D-glucoside	C[C@H]1[C@@H](C[C@H]2[C@H]1[C@@H](OC=C2C(=O)O)O[C@H]1[C@@H]([C@@H]([C@@H]([C@@H](O1)CO[C@H]1[C@@H]([C@@H]([C@@H]([C@@H](O1)CO)O)O)O)O)O)O)O
33.	Minecoside	COc1c(cc(cc1)/C=C/C(=O)O[C@H]1[C@H]2C=CO[C@@H]([C@@H]2[C@@]2([C@H]1O2)CO)O[C@H]1[C@@H]([C@@H]([C@@H]([C@@H](O1)CO)O)O)O)O
34.	Mussaenoside	C[C@@]1(CC[C@H]2[C@@H]1[C@@H](OC=C2C(=O)OC)O[C@H]1[C@@H]([C@H]([C@@H]([C@H](O1)CO)O)O)O)O
35.	Ninpogenin	C1CO[C@@H]2[C@H]1[C@@H](C(=C2)CO)CO
36.	Nuezhenelenoliciside	O=C(OC)C1=CO[C@@H](C)/C(=C\O[C@H]2O[C@@H](CO)[C@@H](O)[C@@H](O)[C@H]2O)/[C@H]1CC(=O)OC[C@H]1O[C@@H](OCCc2ccc(O)cc2)[C@@H](O)[C@@H](O)[C@H]1O
37.	Nuezhenide	C/C=C/1\[C@@H](C(=CO[C@H]1O[C@H]1[C@@H]([C@H]([C@@H]([C@H](O1)CO)O)O)O)C(=O)OC)CC(=O)OC[C@@H]1[C@H]([C@@H]([C@H]([C@@H](O1)OCCc1ccc(cc1)O)O)O)O
38.	Oleoside dimethyl ester	CC=C1C(C(=COC1OC2C(C(C(C(O2)CO)O)O)O)C(=O)OC)C

		C(=O)OC
39.	Oleuropein	C/C=C/1\[C@@H](C(=CO[C@H]1O[C@H]1[C@@H]([C@H]([C@@H]([C@H](O1)CO)O)O)O)C(=O)OC)CC(=O)OCCc1cc(c(cc1)O)O
40.	Patrinalloside A	CC(C)CC(=O)O[C@H]1CC=C(CO[C@H]2O[C@@H](CO)[C@@H](O)[C@@H](O)[C@H]2O)[C@@H]2C[C@@H](O)[C@@H](CO)[C@@H]12
41.	Picroside-II	COc1c(ccc(c1)C(=O)O[C@H]1[C@H]2C=CO[C@@H]([C@@H]2[C@@]2([C@H]1O2)CO)O[C@H]1[C@@H]([C@@H]([C@@H]([C@@H](O1)CO)O)O)O)O
42.	Picroside-III	COc1c(ccc(c1)/C=C/C(=O)OC[C@H]1[C@@H]([C@@H]([C@@H]([C@@H](O1)O[C@H]1[C@@H]2[C@@H](C=CO1)[C@@H]([C@@H]1[C@]2(O1)CO)O)O)O)O)O
43.	Pinnatoside	O=C1C=C(CO[C@H]2O[C@@H](CO)[C@@H](O)[C@@H](O)[C@H]2O)CN1
44.	Plantarenaloside	C[C@@H]1CC[C@]2([C@@H]1[C@@H](OC=C2C=O)O[C@H]1[C@@H]([C@H]([C@@H]([C@H](O1)CO)O)O)O)O
45.	Polystachyn A	C=C(C=O)[C@H]1C(=C(C)C[C@H]1O[C@H]1O[C@@H]2O[C@]3(C)[C@H]1[C@@H](C[C@H]3O)C2=C)C=O
46.	Shanzhiside methyl ester	C[C@@]1(C[C@H]([C@H]2[C@@H]1[C@@H](OC=C2C(=O)OC)O[C@H]1[C@@H]([C@H]([C@@H]([C@H](O1)CO)O)O)O)O)O
47.	Specioside	C1=CO[C@H]([C@H]2[C@@H]1[C@@H]([C@H]1[C@@]2(O1)CO)OC(=O)/C=C/c1ccc(cc1)O)O[C@H]1[C@@H]([C@H]([C@@H]([C@H](O1)CO)O)O)O
48.	Sylvestroside I	OCC[C@H]1CCOC=C1C(=O)O[C@H]1C[C@H]2C(=CO[C@@H](O[C@H]3O[C@@H](CO)[C@@](O)(O[C@H]4O[C@@H](CO)[C@@H](O)[C@@H](O)[C@H]4O)[C@@H](O)[C@H]3O)[C@@H]2[C@H]1C)[C@@H](O)OC
49.	Sylvestroside III	O=C(OC)C1=CO[C@@H](O)[C@H]2[C@H]1C[C@@H](OC(=O)C1=CO[C@@H](O[C@H]3O[C@@H](CO)[C@@H](O)[C@@H](O)[C@H]3O)[C@@H](C=C)[C@H]1CC=O)[C@H]2C
50.	Sylvestroside III dimethyl acetal	O=C(OC)[C@H]1[C@@H]2C[C@@H](OC(=O)C3=CO[C@@H](O[C@H]4O[C@@H](CO)[C@@H](O)[C@@H](O)[C@H]4O)C[C@H]3CC(OC)OC)[C@@H](C)[C@H]2COC1=O
51.	Sylvestroside IV	O=C(OC)C1=CO[C@@H](O)[C@H]2[C@H]1C[C@@H](OC(=O)C1=CO[C@@H](O[C@H]3O[C@@H](CO)[C@@H](O)[C@@H](O)[C@H]3O)[C@@H](C=C)[C@H]1C[C@@H](C)OC)[C@H]2C
52.	Verminoside	C1=CO[C@@H]([C@H]2[C@H]1[C@@H]([C@@H]1[C@]2(O1)CO)OC(=O)/C=C/c1cc(c(cc1)O)O)O[C@H]1[C@@H]([C@@H]([C@@H]([C@@H](O1)CO)O)O)O
53.	Sotorasib	C[C@H]1CN(CCN1c1nc(=O)n(c2nc(c(cc12)F)c1c(cccc1F)O)c1c(ccnc1C(C)C)C)C(=O)C=C
