# Supplementary material for: Establishing the Role of Iridoids as Potential Kirsten Rat Sarcoma Viral Oncogene Homolog G12C Inhibitors Using Molecular Docking; Molecular Docking Simulation; Molecular Mechanics Poisson–Boltzmann Surface Area; Frontier Molecular Orbital Theory; Molecular Electrostatic Potential; and Absorption, Distribution, Metabolism, Excretion, and Toxicity Analysis
Source: Molecules. 2023 Jun 28;28(13):5050. doi: 10.3390/molecules28135050 (PMC10343556; doi:10.3390/molecules28135050)
Supplement: Supplementary file 1 [file molecules-28-05050-s001.zip › Table S2.pdf]

Table S2. Pharmacokinetic behavior of Iridoids and Sotorasib.

Name of the Molecules	MW	TPSA	LogS	LogP	Caco-2 permeability	Pgp inhibitor	Pgp substrate	BBB penetration	CYP 1A2 inhibitor	CYP 2C19 inhibitor	CYP 2C9 inhibitor	CYP 2D6 inhibitor	CYP 3A4 inhibitor	Lipinski rule	Pfizer rule	GSK Rule
6-O-alpha-D-galactopyranosylharpagoside	656.63	254.520	-3.177	-0.352	-6.211	---	+++	--	---	---	---	---	---	Rejected	Accepted	Rejected
6'-O-sinapoyl-geniposide	596.21	199.90	-3.04	1.06	-5.96	---	++	--	---	---	--	-	-	Rejected	Accepted	Rejected
6-O-trans-cinnamoyl-secologanoside	520.16	189.28	-1.85	1.59	-6.22	---	---	---	---	---	---	---	---	Rejected	Accepted	Rejected
6'-O-trans-para-coumaroylgeniposide	536.19	181.44	-2.78	1.03	-5.90	---	--	---	--	---	-	-	-	Rejected	Accepted	Rejected
6'-O-trans-para-coumaroylgeniposidic Acid	522.5	192.440	-2.079	0.651	-6.126	---	+	---	---	---	--	--	--	Rejected	Accepted	Rejected
6-O-trans-p-coumaroyl-8-O-acetylshanzhiside methyl ester	552.52	201.670	-2.289	0.720	-5.955	---	+++	--	---	---	---	--	--	Rejected	Accepted	Rejected
7-hydroxy eucommiol	204.22	101.150	-0.540	-2.003	-5.222	---	---	--	---	---	---	---	---	Accepted	Accepted	Accepted
8-epideoxyloganic acid	360.36	145.910	-1.230	0.069	-6.109	---	--	--	---	---	---	---	---	Accepted	Accepted	Rejected
8-p-coumaroylharpagide	510.49	195.600	-2.876	-0.133	-5.981	---	+++	--	---	---	---	-	-	Rejected	Accepted	Rejected
10-isovaleroyl-dihydropenstemide	528.63	151.980	-4.126	3.332	-4.920	--	++	---	---	---	---	---	---	Accepted	Accepted	Rejected
10-O-acetylgeniposide	430.4	161.210	-1.211	0.498	-5.769	---	---	--	---	---	---	---	---	Accepted	Accepted	Rejected
10-O-succinoylgeniposide	488.44	198.510	-1.279	0.238	-6.058	---	--	-	---	---	---	---	---	Accepted	Accepted	Rejected
Acetylgeniposide	598.55	185.490	-3.074	1.635	-5.209	---	---	---	---	---	---	---	+	Rejected	Accepted	Rejected
Acetylbarlerin	490.46	187.510	-1.595	-0.213	-6.091	---	--	--	---	---	---	---	---	Accepted	Accepted	Rejected
Amphicoside	512.46	197.13	-1.62	-0.73	-6.34	-	+	+	---	---	---	---	---	Rejected	Accepted	Rejected
Asperuloside	416.38	150.210	-1.805	0.267	-6.050	---	+++	--	---	---	---	---	---	Accepted	Accepted	Rejected
Barlerin	448.42	181.440	-1.255	-0.210	-6.190	---	+	--	---	---	---	---	---	Accepted	Accepted	Rejected
Brasoside	358.34	123.910	-1.764	0.788	-5.912	---	+++	--	---	---	---	---	---	Accepted	Accepted	Accepted
Buddlejoside A9	694.72	204.590	-3.598	1.918	-6.203	---	+++	---	---	---	---	---	+	Rejected	Accepted	Rejected
Cantleyoside	746.26	286.89	-0.118	-1.53	-6.12	---	+++	-	---	---	---	---	---	Rejected	Accepted	Rejected
Deacetyl asperuloside	374.34	144.140	-1.364	-0.320	-6.259	---	+++	--	---	---	---	---	---	Accepted	Accepted	Rejected
Euphroside	376.36	166.140	-1.756	-1.325	-6.245	---	++	--	---	---	---	---	---	Accepted	Accepted	Accepted
Eurostoside	492.47	175.370	-2.203	0.650	-5.766	---	+++	--	---	---	---	---	-	Rejected	Accepted	Rejected
Garjasmine	224.21	64.99	-1.11	0.46	-4.72	---	---	+++	---	---	---	---	--	Accepted	Accepted	Accepted
Geniposidic Acid	374.34	166.140	-1.013	-0.640	-6.206	---	---	--	---	---	---	---	---	Accepted	Accepted	Accepted
Gentiopicroside	356.11	134.91	-1.00	-0.906	-5.55	---	---	+	---	---	---	---	---	Accepted	Accepted	Accepted
Isojaslanceoside B	566.55	198.510	-2.318	1.317	-6.120	---	+++	---	---	---	---	---	---	Rejected	Accepted	Rejected
Kutkin	496.16	214.98	-3.95	1.04	-5.64	---	+++	-	--	---	---	---	---	Rejected	Accepted	Rejected
Laciniatoside I	562.56	207.740	-1.419	-0.399	-6.121	---	+++	--	---	---	---	---	---	Rejected	Accepted	Rejected
Laciniatoside II	544.22	201.67	-1.28	-0.57	-6.02	---	++	+	---	---	---	---	---	Rejected	Accepted	Rejected
Loganic acid	376.36	166.140	-0.824	-0.279	-6.389	---	+	--	---	---	---	---	---	Accepted	Accepted	Accepted
Loganic acid 6'-O-beta-D-glucoside	538.19	245.290	-0.786	-1.432	-6.779	---	+++	--	---	---	---	---	---	Rejected	Accepted	Rejected
Minecoside	538.17	197.130	-3.195	0.530	-6.195	---	+++	--	---	---	---	---	-	Rejected	Accepted	Rejected
Mussaenoside	390.38	155.140	-0.997	-0.337	-5.736	---	---	--	---	---	---	---	---	Accepted	Accepted	Accepted
Ninpogenin	170.21	49.690	-0.545	0.020	-4.775	---	---	--	---	---	---	---	---	Accepted	Accepted	Accepted
Nuezhenelenoliciside	686.24	260.59	-1.92	-0.33	-6.14	---	+++	--	---	---	---	---	---	Rejected	Accepted	Rejected
Nuezhenide	686.24	260.59	-1.29	-0.487	-6.15	---	+++	-	---	---	---	---	---	Rejected	Accepted	Rejected
Oleoside dimethyl ester	418.15	161.21	-1.02	-0.24	-5.58	---	+++	+	---	---	---	---	---	Accepted	Accepted	Rejected
Oleuropein	540.51	201.67	-1.57	0.13	-5.84	---	++	+	---	---	---	---	-	Rejected	Accepted	Rejected
Patrinalloside A	460.52	166.140	-2.324	0.272	-5.482	---	+++	--	---	---	---	---	---	Accepted	Accepted	Rejected
Picroside-II	512.46	197.13	-1.62	-0.73	-6.34	---	++	+	---	---	---	---	---	Rejected	Accepted	Rejected
Picroside-III	538.5	197.130	-2.974	0.321	-5.988	---	+++	--	---	---	---	---	--	Rejected	Accepted	Rejected
Pinnatoside	275.26	128.48	-0.75	-2.20	-5.31	---	-	--	---	---	---	---	---	Accepted	Accepted	Rejected

Plantarenaloside	360.36	145.91	-1.90	-0.57	-5.98	---	--	--	---	---	---	---	---	Accepted	Accepted	Rejected
Polystachyn A	360.4	82.06	-3.55	1.16	-5.22	---	+++	--	---	---	---	---	---	Accepted	Accepted	Rejected
Shanzhiside methyl ester	406.38	175.37	-0.91	-0.51	-6.11	---	+	--	---	---	---	---	---	Rejected	Accepted	Rejected
Specioside	508.47	187.90	-3.43	0.43	-6.01	---	+++	--	---	---	---	---	---	Rejected	Accepted	Rejected
Sylvestroside I	748.28	290.05	-0.13	-1.52	-6.18	---	+++	-	---	---	---	---	---	Rejected	Accepted	Rejected
Sylvestroside III	584.57	207.74	-1.59	0.12	-6.03	---	--	--	---	---	---	---	---	Rejected	Accepted	Rejected
Sylvestroside III dimethyl acetal	630.25	209.13	-1.02	-0.33	-5.79	---	-	-	---	---	---	---	---	Rejected	Accepted	Rejected
Sylvestroside IV	614.64	199.90	-1.945	0.679	-5.99	---	--	---	---	---	---	-	+	Rejected	Accepted	Rejected
Verminoside	524.47	208.13	-2.44	0.097	-6.13	---	+++	--	---	---	---	-	+	Rejected	Accepted	Rejected
Sotorasib	560.23	104.45	-5.60	4.43	-4.95	+++	---	---	--	-	+++	++	++	Rejected	Accepted	Rejected

MW: Molecular weight; TPSA: Topological polar surface area;Pgp: P glycoprotein; BBB: Blood brain barrier; CYP: Cytochrome. ---/--: Non inhibitor; +++/++: Inhibitor. -:Non inhibitor with further experiment needed; + : Inhibitor with further experiment needed; BBB^{++}**:** BBB penetrable.
