# Supplementary material for: Establishing the Role of Iridoids as Potential Kirsten Rat Sarcoma Viral Oncogene Homolog G12C Inhibitors Using Molecular Docking; Molecular Docking Simulation; Molecular Mechanics Poisson–Boltzmann Surface Area; Frontier Molecular Orbital Theory; Molecular Electrostatic Potential; and Absorption, Distribution, Metabolism, Excretion, and Toxicity Analysis
Source: Molecules. 2023 Jun 28;28(13):5050. doi: 10.3390/molecules28135050 (PMC10343556; doi:10.3390/molecules28135050)
Supplement: Supplementary file 1 [file molecules-28-05050-s001.zip › Table S3.pdf]

Table S3. Toxicity Profile of Iridoids and Sotorasib.

Name of the Molecules	hERG blocker	Drug Induced Liver Injury	AMES Toxicity	Rat Oral Acute Toxicity	Carcinogenicity	Respiratory Toxicity	Skin Sensitization	Eye Irritation
6-O-alpha-D-galactopyranosylharpagoside	---	-	---	--	--	+++	+	---
6'-O-sinapoyl-geniposide	--	-	--	---	++	+	-	---
6-O-trans-cinnamoyl-secologanoside	---	+++	---	---	+	-	--	---
6'-O-trans-para-coumaroylgeniposide	---	-	-	---	++	--	+	--
6'-O-trans-para-coumaroylgeniposidic Acid	-	++	---	---	++	---	-	---
6-O-trans-p-coumaroyl-8-O-acetylshanzhiside methyl ester	---	++	---	---	++	++	--	---
7-hydroxy eucommiol	---	+++	---	---	--	--	+	+++
8-epideoxyloganic acid	---	++	---	---	++	-	--	---
8-p-coumaroylharpagide	---	--	---	-	++	+++	-	---
10-isovaleroyl-dihydropenstemide	---	++	--	---	+	---	+	---
10-O-acetylgeniposide	---	++	--	--	+++	+++	--	---
10-O-succinoylgeniposide	---	+++	---	---	+++	++	--	---
Acetylgeniposide	---	+++	+++	---	++	++	-	--
Acetylbarlerin	---	++	---	---	++	--	---	---
Amphicoside	---	++	-	+	+	+	---	---
Asperuloside	---	++	+	--	++	++	--	---
Barlerin	---	+	---	---	++	++	---	---
Brasoside	---	++	++	-	++	+++	-	--
Buddlejoside A9	++	+++	-	---	++	+++	+	---
Cantleyoside	+	++	--	+	+++	++	---	---
Deacetyl asperuloside	---	++	-	--	++	++	--	--
Euphroside	---	---	--	---	+++	+++	--	---
Eurostoside	---	+++	+	-	+++	++	-	---
Garjasmine	--	-	+++	+	+++	+++	---	---
Geniposidic Acid	---	+++	---	-	+++	++	--	--
Gentiopicroside	---	--	-	-	+++	---	---	---
Isojaslanceoside B	---	++	---	---	++	-	--	---
Kutkin	---	-	---	---	--	---	++	--
Laciniatoside I	---	--	--	---	-	++	+++	--
Laciniatoside II	---	-	---	++	+++	--	---	---
Loganic acid	---	++	---	---	+++	++	--	---
Loganic acid 6'-O-beta-D-glucoside	---	+++	---	--	-	+	--	---
Minecoside	---	+	--	---	++	+++	++	---
Mussaenoside	---	-	---	---	+++	+++	--	--
Ninpogenin	---	+++	+	+	++	--	-	+++
Nuezhenelenoliciside	---	+++	--	---	+++	--	---	--
Nuezhenide	---	+++	-	---	+++	--	---	---
Oleoside dimethyl ester	---	+++	++	-	+++	+++	---	---
Oleuropein	---	+++	++	--	+++	++	---	---
Patrinalloside A	---	+	---	---	+	---	---	---
Picroside-II	-	++	---	++	++	+	---	---

Picroside-III	-	-	--	---	++	++	-	---
Pinnatoside	---	---	+	---	---	---	+++	--
Plantarenaloside	---	---	--	---	++	++	--	--
Polystachyn A	---	+++	+++	+++	+	+++	++	---
Shanzhiside methyl ester	---	-	---	---	+++	++	---	---
Specioside	---	--	---	--	+	+++	---	---
Sylvestroside I	--	+++	---	++	+++	-	---	---
Sylvestroside III	---	+	+	--	+++	+++	+	---
Sylvestroside III dimethyl acetal	--	++	--	+	+++	++	---	---
Sylvestroside IV	--	++	+	--	+++	+++	--	---
Verminoside	-	+	--	--	++	---	++	---
Sotorasib	+	+++	---	--	++	++	---	---

+++: Highly toxic; ++: Toxic; ---: Non toxic; --: Non toxic; - : Non toxic with further experiment needed; + : Toxic with further experiment needed.
