# Supplementary figures and images for: Establishing the Role of Iridoids as Potential Kirsten Rat Sarcoma Viral Oncogene Homolog G12C Inhibitors Using Molecular Docking; Molecular Docking Simulation; Molecular Mechanics Poisson–Boltzmann Surface Area; Frontier Molecular Orbital Theory; Molecular Electrostatic Potential; and Absorption, Distribution, Metabolism, Excretion, and Toxicity Analysis
Source: Molecules. 2023 Jun 28;28(13):5050. doi: 10.3390/molecules28135050 (PMC10343556; doi:10.3390/molecules28135050)

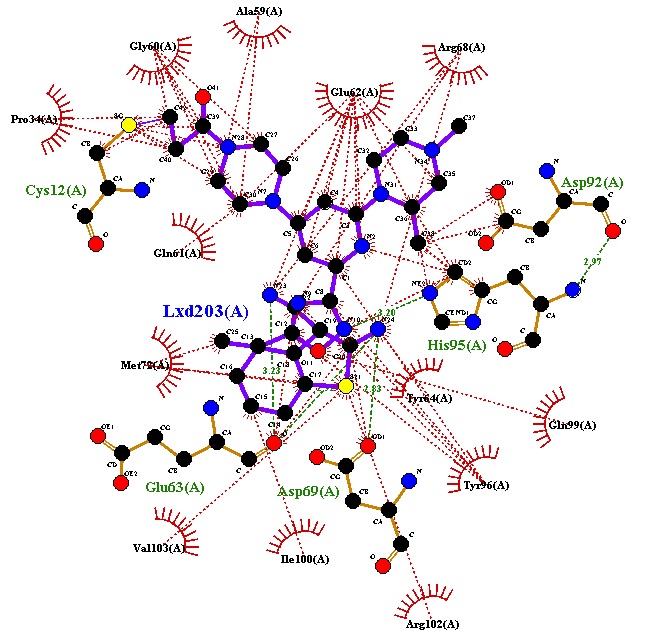

Supplement: Supplementary file 1 [file molecules-28-05050-s001.zip › Figure S1.jpg]

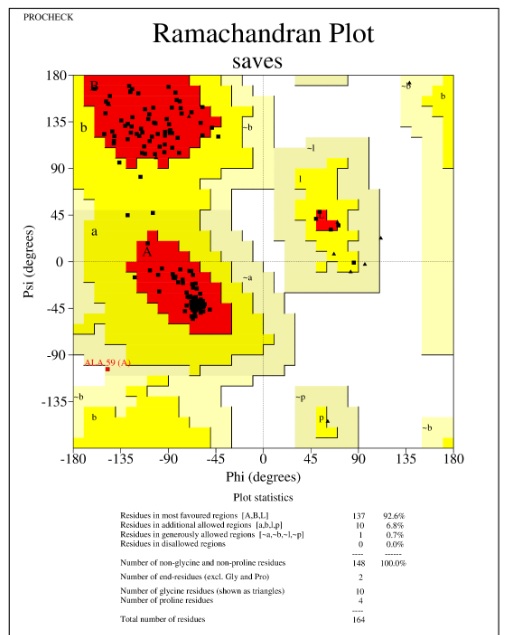

Supplement: Supplementary file 1 [file molecules-28-05050-s001.zip › Figure S2.jpg]
